# Development of a ParticipACTION App–Based Intervention for Improving Postsecondary Students’ 24-Hour Movement Guideline Behaviors: Protocol for the Application of Intervention Mapping

**DOI:** 10.2196/39977

**Published:** 2023-03-14

**Authors:** Stephanie M Flood, Brooke Thompson, Guy Faulkner, Leigh M Vanderloo, Beth Blackett, Matt Dolf, Amy E Latimer-Cheung, Mary Duggan, Katie M Di Sebastiano, Kirstin N Lane, Melissa C Brouwers, Julia McKenna, Tala Chulak-Bozzer, Daniel Fuller, Geralyn R Ruissen, Shelby L Sturrock, Jennifer R Tomasone

**Affiliations:** 1 School of Kinesiology and Health Studies Queen's University Kingston, ON Canada; 2 School of Kinesiology University of British Columbia Vancouver, BC Canada; 3 Department of Science and Evaluation ParticipACTION Toronto, ON Canada; 4 Student Wellness Services Queen's University Kingston, ON Canada; 5 Office of Wellbeing Strategy University of British Columbia Vancouver, BC Canada; 6 Canadian Society for Exercise Physiology Ottawa, ON Canada; 7 Department of Sport and Exercise Sciences Durham University Durham United Kingdom; 8 School of Exercise Science Physical and Health Education University of Victoria Victoria, BC Canada; 9 School of Epidemiology and Public Health Faculty of Medicine University of Ottawa Ottawa, ON Canada; 10 Department of Community Health and Epidemiology College of Medicine University of Saskatchewan Saskatoon, SK Canada; 11 Faculty of Kinesiology, Sport, and Recreation University of Alberta Edmonton, AB Canada; 12 Division of Epidemiology Dalla Lana School of Public Health University of Toronto Toronto, ON Canada

**Keywords:** 24-Hour Movement Guidelines, multiple behavior change, mobile health, mHealth, postsecondary students, physical activity, sedentary behavior, sleep, app, mobile phone

## Abstract

**Background:**

The Canadian 24-Hour Movement Guidelines for adults provide specific recommendations for levels of physical activity, sedentary behavior, and sleep (ie, the movement behaviors) required for optimal health. Performance of the movement behaviors is associated with improved mental well-being. However, most postsecondary students do not meet the movement behavior recommendations within the Canadian 24-Hour Movement Guidelines and experience increased stress and declining well-being, suggesting the need for an intervention targeting students’ movement behaviors.

**Objective:**

We aimed to develop and implement a theory-informed intervention intended to improve the movement behaviors and mental well-being of first-year postsecondary students.

**Methods:**

The Intervention Mapping protocol was applied in the development and implementation of the intervention. Intervention Mapping entailed performing a needs assessment, determining the intervention outcomes, selecting theory- and evidence-based change methods and applications, preparing and producing intervention plans and materials, developing the implementation plan, and finally developing an evaluation plan. The Theoretical Domains Framework and the Behavior Change Wheel were also used in conjunction with the Intervention Mapping protocol to ensure a solid theoretical basis for the intervention. This protocol led to the development and implementation of a 6-week, theory-informed ParticipACTION app–based intervention aimed at helping first-year postsecondary students improve their movement behaviors and mental well-being. The developed app content provided students with information on each of the movement behaviors and behavioral strategies (ie, goal setting, action planning, monitoring, and coping planning). The use of Intervention Mapping allowed for the continuous involvement of various multidisciplinary partners and end users, ensuring that the intervention design and implementation was appropriate for the target audience. The feasibility, acceptability, and potential impact of the intervention will be examined in a subsequent proof-of-concept study at 2 Canadian university campuses.

**Results:**

Participant recruitment occurred during September 2021, and the intervention was conducted from October to December 2021. The deadline for completion of the postintervention questionnaire by participants was mid-December 2021. The analysis of data examining the feasibility, acceptability, and potential impact of the intervention began in January 2022, with the publication of the proof-of-concept evaluation expected in 2023.

**Conclusions:**

Intervention Mapping with the Theoretical Domains Framework and Behavior Change Wheel was a useful approach to combine evidence and theoretical concepts to guide the design and implementation of a ParticipACTION app–based intervention targeting postsecondary students’ movement behaviors and mental well-being. This process may serve as an example for other researchers developing multiple behavior change app–based interventions. Should the forthcoming evaluation demonstrate the intervention’s acceptability, feasibility, and potential impact, the intervention may provide a scalable method of improving postsecondary students’ movement behaviors and mental well-being.

**International Registered Report Identifier (IRRID):**

RR1-10.2196/39977

## Introduction

The Canadian 24-Hour Movement Guidelines (24HMG) for adults aged 18 to 64 years and adults aged 65 years or older [1] provide specific recommendations for ideal levels of physical activity, sedentary behavior, and sleep required for optimal health. Despite the established physical and mental health benefits of increasing physical activity, decreasing sedentary behavior, and ensuring sufficient sleep [1], population-level engagement in these behaviors is suboptimal [2]. Indeed, although a guideline can signal the recommended action, it alone does not guarantee use [3]. As a result, purposefully chosen and implemented evidence-informed efforts to increase the uptake and use (ie, implementation) [4] of the 24HMG among target populations are required.

To address this gap and using an integrated knowledge translation approach [5,6] and the Knowledge-to-Action Framework [6] as an organizing model, the developers of the 24HMG established a knowledge translation team [7] comprising partners (eg, representatives from the Canadian Health Promoting Campuses Network, Canadian Sleep Society, Canadian Medical Association, and Canadian Public Health Association) and researchers who together designed, implemented, and evaluated a strategy to promote adoption of the 24HMG.

The knowledge translation team identified postsecondary students (ie, students entering or attending college or university, also known as *tertiary education institutions* internationally) as the target audience for guideline implementation (see the study by Tomasone et al [7]). Despite the evidence supporting the link between movement behaviors and impacts on their mental health and well-being [8,9] and academic performance [10], most postsecondary students engage in suboptimal levels of the movement behaviors [9]. Moreover, the COVID-19 pandemic exacerbated students’ suboptimal movement levels with stay-at-home orders and widespread remote learning [11-14]. Short-term negative consequences of this change included increased stress and declining mental health and well-being (eg, higher rates of severe anxiety) [15,16]. The long-term impact on students’ physical and mental health remains unknown. Thus, an intervention targeting the movement behaviors and mental well-being of postsecondary students was timely and necessary.

Interventions developed using theory may be more effective than those based on little or no use of theory [17]. To inform intervention design and implementation, we applied Intervention Mapping—a rigorous and systematic protocol for developing theory- and evidence-based behavior change interventions [18,19]. Although Intervention Mapping offers an iterative protocol for developing and tailoring an implementation intervention, it was necessary to use other frameworks in conjunction with Intervention Mapping to fully inform design and implementation. The Theoretical Domains Framework (TDF) and Behavior Change Wheel (BCW) were used to help inform the Intervention Mapping protocol. The TDF is a theoretical framework that provides a way to view the cognitive, affective, social, and environmental influences on behavior [20] and maps onto the capability, opportunity, motivation, behavior (COM-B) model within the BCW. At its core, the BCW has the model of behavior known as COM-B, referring to “capability,” “opportunity,” “motivation,” and “behavior,” which recognizes that behavior is part of an interacting system involving all these components [21,22]. The BCW identifies different intervention options that can be applied to change each of the components and policies that can be adopted to deliver those intervention options [21,22]. Finally, the BCW involves identifying content and implementation options (ie, identifying behavior change techniques [BCTs] and modes of delivery) [21,22]. Given the complexity of applying these 2 frameworks in conjunction with the Intervention Mapping protocol, this paper provides a systematic description of the process used to design and implement an intervention aimed at improving the movement behaviors and well-being of first-year postsecondary students (ie, students who have just graduated high school or took a single gap year before entering an undergraduate program and turning 18 years or older).

## Methods

### Overview

The Intervention Mapping protocol specifies 6 steps for the development of theory and evidence-based interventions [18,19]. Step 1 (the needs assessment) involves establishing a planning or working group, defining the context of the intervention in terms of the health problem and population (ie, intervention target group), establishing the determinants of behavior change (ie, the barriers and facilitators), and finally describing the intervention setting. In step 2, the intervention outcomes are determined, which include performance objectives (ie, behavioral changes necessary to achieve intervention outcomes) and change objectives (ie, specific behaviors that will lead to changes in the behavioral determinants, which are needed to achieve performance objectives). Step 3 involves choosing theory- and evidence-based change methods, selecting or designing practical applications to deliver change methods, and finally refinement of the selected intervention components. Step 4 focuses on intervention production by preparing intervention plans and drafting protocols and materials followed by the pretesting, refinement, and production of intervention materials. In step 5, the development of an intervention implementation plan occurs and includes identifying intervention program users (ie, adopters and implementers). Step 6 comprises specifying an evaluation design and developing the indicators and measures of assessment.

**Figure 1:**
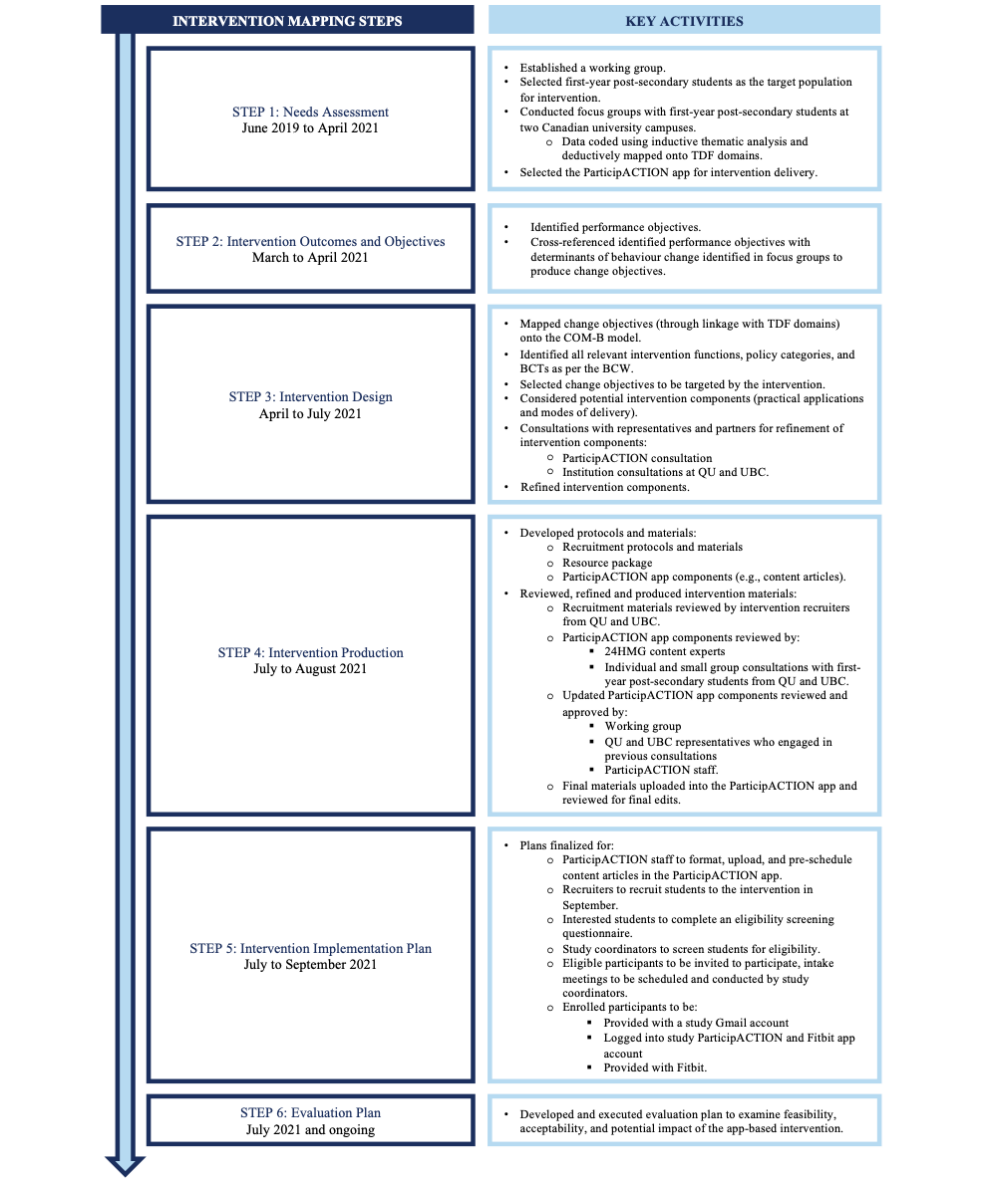
Timeline displaying the 6 Intervention Mapping steps and key activities within each step. 24HMG: 24-Hour Movement Guidelines; BCW: Behavior Change Wheel; COM-B: capability, opportunity, motivation, and behavior; QU: Queen’s University; TDF: Theoretical Domains Framework; UBC: University of British Columbia.

We implemented the first 5 steps in the development and implementation of the intervention. Step 6 (evaluation plan) will be published in detail in a forthcoming proof-of-concept evaluation paper; however, a brief synopsis of the evaluation plan is described in subsequent sections. Figure 1 outlines the 6 Intervention Mapping steps, the period over which each step was performed, and the key activities within each step.

### Step 1: Needs Assessment (June 2019-April 2021)

#### Establish a Working Group

We formed a multidisciplinary team of partners and researchers to create a new working group (see Multimedia Appendix 1 for the working group constituency and roles). Several working group members were members of the original 24HMG Knowledge Translation Advisory Committee [7], and additional members were added to ensure comprehensive expertise for intervention development, implementation, and evaluation. The working group collectively possessed expertise in the 24HMG behaviors, tailored intervention development and delivery, postsecondary student health and well-being, and knowledge translation and behavior change research and evaluation. Working group members were engaged at various steps of the Intervention Mapping approach.

#### Description of Health Problem and Intervention Target Group

Three factors contributed to defining the health problem and intervention target group: (1) established, suboptimal performance of the movement behaviors (ie, not meeting 24HMG recommendations); (2) suitability of the intervention target group for receiving a targeted intervention; and (3) adoption of a health-promoting charter directing action at postsecondary institutions.

Postsecondary students are at risk of negative physical and mental health impacts associated with suboptimal performance of the movement behaviors. In the first deployment of the Canadian Campus Wellbeing Survey in early 2020, Weatherson et al [9] reported that only 9.9% of postsecondary students achieved 4 components of the 24HMG (moderate to vigorous physical activity, total sitting time, recreational screen time, and sleep). Large decreases in physical activity levels, sleep quantity, and sleep quality as well as increases in students’ sedentary behavior are observed as students (adults aged 18-19 years) adjust to the transition from high school to postsecondary education [23-28]. This life transition, compounded with the negative impact of the COVID-19 pandemic on students’ movement behaviors [11-14], may further negatively impact students’ short- and long-term physical and mental health.

Fortunately, postsecondary students are malleable learners and an intervention that provides an opportunity for students to learn and develop healthy movement behaviors, especially in their first year, can facilitate the development of habits that they can continue to use in their adult years ahead [29]. In addition, physical activity and sleep have been found to be positively associated with postsecondary students’ positive affect and mental health [30,31], suggesting further benefit of postsecondary students receiving this intervention.

Several Canadian postsecondary institutions have adopted the Okanagan Charter for Health Promoting Universities and Colleges [32]. The Okanagan Charter calls on institutions to embed health in all aspects of campus culture and to lead health promotion action and collaboration. The working group anticipated that campuses that have adopted the charter would be supportive in helping to develop, implement, and evaluate an intervention aimed at improving students’ movement behaviors and mental well-being. Two Canadian campuses—Queen’s University (QU) and the University of British Columbia (UBC)—were selected as sites for implementation of a fall 2021 intervention, as both institutions have adopted the charter and had 24HMG knowledge translation team member representation on these campuses (ie, team members GF and MD are located at UBC, while JRT, AELC, and SMF are located at QU).

#### Determinants of Behavior Change

Given the novelty of the 24HMG in Canada, there was a paucity of published data on the determinants (ie, barriers and facilitators) of postsecondary student-integrated movement behaviors; thus, we collected our own. Focus groups were conducted with 74 first-year undergraduate postsecondary students at QU and UBC [33]. Focus group data were coded using inductive thematic analysis before codes were deductively mapped onto TDF domains [34,35]. The TDF was used to more thoroughly understand the individual-level factors that may act as barriers or facilitators to behavior change; within Intervention Mapping, the TDF domains represent the determinants of behavior change. In total, 13 of the 14 TDF domains were identified as influencing students’ movement behaviors, with the unidentified TDF domain being *optimism*. For each code within every TDF domain, a belief statement was generated to provide details about the perceived role of the domain in influencing the behavior [36], helping to exemplify the barriers and facilitators experienced by students when attempting to engage in the movement behaviors [33]. Multimedia Appendix 2 [21,22,33] provides a full list of barrier and facilitator belief statements and change objectives.

Beliefs within the *Knowledge* and *Behavioural Regulation* domains were cited as facilitators and those within the *Social/Professional Role* and *Identity* domains were cited as barriers to engaging in guideline-level movement behavior. Beliefs within 10 domains (*Skills; Beliefs about Capabilities; Beliefs about Consequences; Reinforcement; Intentions; Goals; Memory, Attention and Decision Processes; Environmental Context and Resources; Social Influences;* and *Emotions*) were cited as both facilitators and barriers to students performing the movement behaviors. Interestingly, many of the determinants operate at an individual level (eg, within the students’ control, such as developing and maintaining a schedule), whereas other determinants operate beyond the students’ immediate control (eg, the university’s provision of standing desks and desk cycles for student use). See Multimedia Appendix 2 for more details.

#### Intervention Setting

Intervention development occurred during the COVID-19 pandemic; consequently, there was uncertainty about the timing of a return to campus. To minimize in-person intervention components, using an app was appealing as an intervention delivery mechanism. An app is a useful vehicle for intervention delivery for this population, as students tend to report high use of health-related apps [37,38]. Although an app-based intervention is restricted to only being able to target individual-level determinants of behavior change, given the unpredictability of the COVID-19 pandemic, opting for an app-based delivery was a pragmatic decision. Not only are app-based interventions a viable means of intervention in the target population, but previous empirical evidence has also demonstrated that apps can be effective in changing health-related cognitions and behavior [39-41].

ParticipACTION, a physical activity nonprofit organization and one of the main partners of the 24HMG, already possessed an evidence-informed app that was leveraged by the working group to help support and expedite the implementation of the intervention within a short timeframe. The ParticipACTION app was developed using the BCW framework [21,22] as its theoretical basis [42].

The free ParticipACTION app serves as both a self-monitoring device (tracking users’ steps, “move minutes” and “active minutes” by linking the app with a fitness tracker [eg, Fitbit or Apple Watch] or having users manually add their activity) as well as a content hub (where evidence-informed articles are provided to help educate users on the 24HMG, goal setting, barrier identification and action planning)—these components work together to provide a tailored experience for users. App users are also eligible to win rewards (eg, weekly draw for a CAD $10 [US $7.4] Amazon gift card funded by ParticipACTION and its funders: the Public Health Agency of Canada and Sports Canada) by earning daily badges (ie, extrinsic motivation), where each badge releases entries in the next draw for rewards automatically. The app user earns additional badges by tracking their activity and hitting their active minute goals, engaging in challenges, reading articles, and watching videos within the app. ParticipACTION also offers “ParticipACTION+,” a premium for-a-fee version that includes all standard app features as well as additional features such as in-app team challenges and leaderboards (eg, to compare step counts against other organization or team members), member engagement reporting, and customizable content and notification delivery. Fees for ParticipACTION+ are paid for by the intervention delivery team and not by app users. As the ParticipACTION app was theoretically informed and would allow for us to address many of the identified determinants of behavior change without in-person contact, it was deemed an appropriate vehicle for intervention delivery.

### Step 2: Intervention Outcomes and Objectives (March 2021-April 2021)

The primary intervention outcome was to improve first-year undergraduate postsecondary students’ movement behaviors, whereas the secondary outcome was to improve students’ mental well-being. The performance objectives for students’ movement behaviors are listed in Multimedia Appendix 2 and were drawn from each of the main 24HMG recommendations (ie, physical activity, sedentary behavior, sleep, and integration of the behaviors). Performance objectives were cross-referenced with the belief statements (ie, determinants) generated in step 1 (section *Determinants of Behaviour Change*) to produce the change objectives in Multimedia Appendix 2. The change objectives state what needed to change about a specific behavioral determinant (eg, intentions) for students to achieve a specific performance objective. In some instances, multiple belief statements were collapsed together as they represented a common change objective. Multimedia Appendix 2 includes all the performance objectives and corresponding potential change objectives identified from the focus group data.

In line with the secondary outcome of improving students’ mental well-being, a final performance objective was identified: “student has positive feeling and functional aspects of mental well-being (eg, feeling optimistic, having energy to spare).” Given that following the 24HMG is associated with a lower risk of anxiety and depression and improved quality of life [1], the working group interpreted the change objectives for this final performance objective as students’ performance of the movement behaviors themselves.

### Step 3: Intervention Design (April 2021-July 2021)

#### Theoretical Methods

To move forward with intervention design and selecting change methods for step 3, we examined the theoretical links between the generated belief statements (see the section Step 1: Needs Assessment [June 2019 to April 2021]) organized by TDF domain and the antecedents of behavior, which can be selectively targeted in an intervention. Through their linkage with the TDF domains, change objectives were mapped onto the COM-B model, and accordingly, all relevant intervention functions and policy categories were identified following the process described in the BCW guide [22] (Multimedia Appendix 2). The next step was to identify intervention content in terms of which BCTs best serve the intervention functions. BCTs are considered the “active ingredients” of an intervention designed to change behavior and are the proposed mechanism of change to be used alone or in combination with other BCTs [43]. To assist in selecting candidate BCTs for the intervention, we used the Theory and Techniques Tool [44] to specify the links between TDF domains and BCTs [44-46].

Members of the working group reviewed Multimedia Appendix 2 (ie, the complete list of formulated change objectives mapped onto the TDF and BCW) and considered which change objectives could and should be addressed within the app-based intervention. The discussion included reflecting on the identified TDF domains, COM-B sources of behavior, intervention functions, policy categories, and BCTs. Selected change objectives are set in bold in Multimedia Appendix 2. Working group members believed that all 6 sources of behavior change (psychological and physical capabilities, social and physical opportunities, and automatic and reflective motivation) and 13 of the 14 TDF domains should be targeted within the intervention.

Notably, all selected change objectives focus on individual-level changes, with organizational- and community-level changes lying outside the scope of an app-based intervention. However, Multimedia Appendix 2 outlines all the identified change objectives, where some change objectives address organizational-level changes. One example is “encouraging residences to enforce quiet hours and promote an atmosphere that encourage healthy sleep habits.”

#### Intervention Components: Practical Applications and Mode of Delivery

##### Overview

Multimedia Appendix 3 presents the intervention components described in this section as well as a description of the evidence and rationale that guided the formation of each component. In total, 7 out of the 9 possible BCW intervention functions were selected: education, persuasion, incentivization, training, enablement, modeling, and environmental restructuring. To support implementation of the intervention functions, 2 BCW policy categories were selected: communication and marketing and environment and social planning.

The working group considered the potential intervention components (ie, practical applications and modes of delivery) to facilitate the identified change methods. The BCW intervention functions and policy categories guided the selection of the intervention components. In addition, the BCW presents the affordability, practicality, effectiveness, acceptability, safety, and equity criteria, which should be applied when engaging in the process of developing, selecting, and implementing interventions [22]; the working group reflected on these criteria during the component selection process. Five intervention components, described in subsequent sections, were considered to facilitate change objectives: ParticipACTION app content articles, notifications, badges, and competition as well as a resource package outside of the ParticipACTION app.

##### ParticipACTION App: Content Articles

A series of content articles specific to the 24HMG would be created, tailored for first-year postsecondary students, and uploaded into the ParticipACTION app. These articles would take student participants on a “content journey,” providing information about each of the individual movement behaviors and how the movement behaviors are connected, while also addressing related barriers and facilitators identified in the needs assessment. For example, content would include information about the benefits of performing the movement behaviors pertinent to postsecondary students. Articles would also persuade and enable participants to perform the movement behaviors by focusing on select BCTs (eg, action planning). Articles would provide suggestions on how to restructure physical and social environments to encourage performance of the movement behaviors (eg, improvising a standing desk and how to be active with roommates). Students would be able to visit articles multiple times if desired, and through ParticipACTION+, articles could appear on students’ app home page. Although students would have the flexibility to explore the ParticipACTION app, they would be presented with the developed content articles sequentially over the intervention period.

##### ParticipACTION App: Notifications

The use of the ParticipACTION+ service would enable the development and delivery of both push notifications and movement behavior reminder notifications. Push notifications would inform participants of the release of new content article in the app and would link to the new article. Movement behavior reminder notifications would encourage participants to engage in a specific movement behavior. These reminder notifications would serve as a prompt for the behavior, which was identified as an important BCT.

##### ParticipACTION App: Content Article Engagement Badges

The development of a weekly content article engagement badge was proposed because ParticipACTION app users can already earn badges for the number of articles read and their physical activity (eg, number of steps). Participants would earn a badge for engaging with the intervention content articles each week. The allocation of badges to participants would provide general encouragement to participants, reinforcing their behavior of engaging with the content articles.

##### ParticipACTION App: Competition

Competition was identified within the needs assessment as a facilitator for movement behavior engagement and would offer students a form of social comparison. The working group proposed 2 types of competition: within-campus and between-campus competitions. For within-campus competition, participants attending the same institution would be placed on teams that would compete to see which team has the highest step count or the most move minutes using the ParticipACTION+ challenge app feature. Participants would be able to view scores on a leaderboard to determine how other teams were performing. A reward would be offered where participants on the winning team would each win a material reward (eg, e-gift card) at the end of the intervention. Between-campus competition would involve participants at each institution competing to see which institution can collectively accumulate more move minutes by the end of the intervention. A weekly notification would be used to inform participants of their institution’s progress. Similar to the within-campus competition, participants at the winning institution would each receive a material reward (eg, e-gift card) at the end of the intervention.

##### Resource Package

Formative work identified that participants needed access to resources on their campus or within the city to assist with meeting the behaviors, including free and low-cost options. Rather than providing tailored resources for each institution (ie, QU and UBC) and its respective city (ie, Kingston, Ontario, and Vancouver, British Columbia) within the app content, the working group proposed developing a tailored resource package that would be shared with participants via participant email. This decision allowed for the developed app content to be applicable to both campuses rather than requiring 2 sets of app content tailored to each institution’s campus and city, supporting future scalability of the intervention.

##### Nonintervention Components

The working group acknowledged that by giving students access to the ParticipACTION app, they would have access to the additional ParticipACTION app components (eg, “Take 10” prompts, exercise videos, and rewards). Students would not be instructed to explicitly use these additional components as part of the intervention, but they might be of benefit to those students who engaged with these additional ParticipACTION app components. In addition, the working group decided a commercial wearable device (ie, the Fitbit Inspire 2) would be provided to participants as a device-assessed measure of their movement behaviors. Fitbit was chosen instead of researcher-grade devices (eg, accelerometer) for several reasons. First, the physical activity data from Fitbit would be synced into the ParticipACTION app. Accordingly, all participants would have equal access to the full app functionality (ie, monitoring steps and active minutes directly within the ParticipACTION app). Second, because our interest was in all movement behaviors reported over a 24-hour period, we determined that using a device-assessed measure of movement behaviors would decrease participant burden and increase the accuracy of movement behaviors compared with self-report measures [47,48]. Finally, wrist-worn consumer-grade wearable devices increase consumer appeal and wear compliance compared with waist-worn research-grade accelerometers [47,49]; thus, we hoped that this would translate into increased compliance among our target population.

It is conceivable that Fitbit alone could have provided participants self-monitoring and feedback. However, though Fitbits (and other consumer wearables) have been linked with multiple theoretically relevant strategies associated with increased adherence and maintenance of movement behaviors such as physical activity [50], when wearable technology alone has been given to participants, without any intervention component, minimal changes in physical activity behavior are observed [51]. Consequently, Fitbit itself would not be considered an intervention component.

Considering all the above components, the working group proposed an intervention length of 6 weeks during the second half of postsecondary students’ 12-week fall 2021 term, allowing sufficient time for intervention recruitment and intake while ensuring the completion of the intervention before end-of-term examinations. The working group proposed that limiting content articles to 2 succinct articles per week for the 6-week intervention for a total of 12 articles would prevent overwhelming students and may help to potentially circumvent intervention attrition. On the basis of the formative work conducted, the format of the proposed content journey would be such that the first article each week would be “informational” in nature, providing necessary knowledge with tips and benefits pertinent to participants, and the second article for each would focus on a particular behavior change strategy, describing how to perform the behavior change strategy in the context of one specific movement behavior.

#### Refining Intervention Components

Consultations with the broader ParticipACTION team and partners at both institutions were held to obtain further insight on the affordability, practicality, effectiveness, acceptability, safety, and equity of the proposed intervention. Engaging these additional partners allowed for the selection and further refinement of the proposed intervention components.

##### ParticipACTION Review

Although ParticipACTION representatives sit on the working group (Multimedia Appendix 1), broader feedback was sought from additional ParticipACTION representatives on the feasibility of the proposed intervention components. A written draft of the components was reviewed by ParticipACTION staff with knowledge in the following areas: behavior and insights, knowledge translation, marketing and communications, product marketing, strategy and insights, and health promotion. ParticipACTION staff supported the inclusion of all the proposed components with the exclusion of the content article engagement badges. Reasoning for exclusion of this component was owing to feasibility related to the creation of this app badge within the intervention development timeframe and budget. Accordingly, the working group agreed to exclude this intervention component.

##### Institution Representative Consultations

Consultations were held with representatives (ie, campus staff and students) from each institution using Zoom (Zoom Video Communications). The consultation at QU was held with 9 representatives from Student Wellness Services, Residence Life, Q Success (ie, a first-year peer mentorship program), the International Centre (ie, cross-cultural advisory), and first-year postsecondary students attending QU. The UBC consultation was held with 14 members of the campus Physical Activity Committee, which included individuals from Athletics and Recreation, the Office of Wellbeing Strategy, the University Neighbourhoods Association, the Student Wellness Centre, Kinesiology Undergraduate Society, and the Graduate Student Society.

Before the consultations, representatives were provided with a description of the intervention and potential evaluation components, including participant eligibility and recruitment, intervention components (eg, proposed content for the articles), and proposed evaluation components. During group-based, 60-minute consultations via Zoom, facilitated by SMF and JRT, representatives were presented with the findings of the formative research conducted as part of the needs assessment. Group discussion focused on obtaining representatives’ insight on the “content journey” articles (eg, order of movement behavior content, behavior change strategies, and frequency of content) and inclusion of the competition feature of the app. Representatives were then asked to provide feedback on the proposed participant eligibility (ie, inclusion and exclusion criteria) to suggest potential avenues and strategies for intervention recruitment and to comment on proposed evaluation components. Representatives’ suggestions from both consultations were summarized and brought forward to the working group for discussion.

Representatives agreed with the proposed intervention length of 6 weeks, to limiting content to 2 articles per week, and to the format of the content journey. However, the main topic of discussion during the consultations was about the order of presentation of the movement behaviors within the content journey articles. Originally, the working group proposed focusing on behaviors in the order presented in the 24HMG tag line of “Move More, Reduce Sedentary Time, Sleep Well” (ie, physical activity, sedentary behavior, and sleep). However, representatives reported that many students are aware that they are not obtaining sufficient physical activity; rather than immediately focusing on a behavior that individuals often perceive as being difficult to perform, it may be more motivating to start with a behavior they may perceive as being easier to achieve. With this feedback, the working group altered the content journey to begin by introducing the 24HMG, then present content focused on the movement behaviors in sequence from sleep, sedentary behavior, light physical activity, to moderate to vigorous physical activity, and finally revisiting the integrated nature of the movement behaviors at the end of the intervention.

Finally, representatives advocated for the removal of the competition component of the intervention. The ParticipACTION app does not offer team communication as part of its functionality; given that social connection and support would be important for successful between- and within-campus competition and maintaining participant engagement in competition, the working group agreed to remove this intervention component. See Multimedia Appendix 3 for detailed descriptions of the intervention components (both included and excluded, following representative consultation).

### Step 4: Intervention Production (July 2021-August 2021)

#### Prepare Plans and Draft Protocols and Materials

With the input of our working group and the application of feedback from the consultations conducted in step 3, we moved forward with outlining the participant recruitment protocols and materials for each institution, the resource package, app content articles, and app notifications.

##### Recruitment Protocol and Materials

Participant recruitment serves as the vehicle of entry for participants into the intervention; thus, recruitment protocol and materials were carefully considered. It was determined that participant recruitment would involve student self-referral to the intervention to limit the burden on recruiters (ie, campus representatives who would be asked to assist with recruitment). At QU and UBC, recruitment avenues included residence advisors or counselors, Student Wellness Services, and undergraduate societies and programs. Recruiters were provided with a 2-page study description as well as posters and preprepared social media posts. Recruiters were asked to place study posters in areas that students were likely to view (eg, student spaces in residences, wellness services, or office bulletin boards) and use preprepared social media posts on their institutional office, service, or society social media accounts (ie, Facebook, Instagram, and Twitter). In addition, instructors of first-year courses at each institution were contacted and provided with a Microsoft PowerPoint slide to add to the beginning of their lecture presentations as well as brief intervention descriptions they could post on their course pages on their institutional course platforms. All developed recruitment materials included links and QR codes for the intervention eligibility screening questionnaire for interested students to complete.

##### Resource Package

The resource package, a PDF document that would be emailed to participants, was developed by the 24HMG project coordinator (SMF) and research assistant (BT) to include the public facing 24HMG; infographics (ie, tips to “move more, reduce sedentary time, sleep well,” benefits of following the 24HMG); and physical activity, sedentary behavior, sleep, and mental well-being resources for campus, community, and web. The resource packages were tailored for each campus and institution city with input from QU Student Wellness Services and UBC Wellbeing.

##### ParticipACTION App Components

ParticipACTION shared existing app content (ie, evidence-based articles created by ParticipACTION and partners) on the topics of instruction, lifestyle, and behavior change. A research assistant (BT)—a fourth-year undergraduate kinesiology student at QU trained in behavior change theory and practice—reviewed the existing ParticipACTION app content, selected intervention functions, and BCTs (Multimedia Appendix 3) to draft the 12 content journey articles. In total, 12 accompanying push notifications (ie, to notify participants about each new piece of content in the app) and 12 behavior reminder notifications (ie, to remind participants to engage in movement behaviors) were simultaneously drafted to ensure consistent messaging with the content articles.

The developed content articles followed the order of behaviors and behavior strategies defined and selected in step 3 (see Multimedia Appendix 3 for the description of the content articles). Notifications addressing the same movement behavior were scheduled to be staggered across the 6-week period to emphasize the integrated nature of the behaviors. An iterative content review process with the 24HMG project coordinator (SMF) and knowledge translation lead (JRT) was undertaken.

#### Pretest, Refine, and Produce Materials

##### Recruitment Materials

The developed recruitment materials were shared with recruiters from QU (Student Wellness Services and Residence Life) and UBC (UBC Wellbeing) for feedback. Minor feedback was provided on the social media posts, suggesting that the amount of text be reduced.

##### Resource Package

The resource package was sent to QU and UBC campus representatives on the working group who collaborated with colleagues on their end to provide additional suggestions on the developed resources. The final version was reviewed by a subset of the working group and approved for distribution.

##### ParticipACTION App Components

The developed content journey articles and notifications were reviewed by the 24HMG content experts (ie, researchers involved in the development of the 24HMG recommendations). Incorporated feedback from content experts included (1) making the language less formal, (2) distinguishing more clearly between sedentary time and screen time, (3) hyperlinking to existing ParticipACTION app content (ie, exercise videos) within the content articles, and (4) adding calls to action (eg, “What is your SMART goal?”) within the content articles.

Individual and small group consultations were subsequently held with 9 first-year postsecondary students across both institutions, facilitated by the 24HMG research assistant (BT). Students were sent Microsoft Word document drafts of the content articles and notifications in advance of the consultations. During consultations held via Zoom, students were asked to consider whether first-year students would find the content engaging, motivating, exciting, appropriate, and interesting and whether the content was too long or too short. Students were also encouraged to provide feedback on phrasing and whether additional tips or strategies should be included. Feedback from students that was used to refine content included (1) breaking down bigger paragraphs to bullet points for visual appeal, (2) making the content articles read as conversational with the use of rhetorical questions, (3) adding definitions for terms that are less known (eg, sedentary behavior and screen time), (4) adding yoga to the coping planning behavior change strategy article (a popular activity), and (5) breaking down the 150 minutes of moderate to vigorous physical activity into more “manageable” chunks (eg, five 30-minute segments).

Students were also queried about which days of the week and time of day they would prefer to receive app notifications; they reported a preference for push notifications (for content articles) in the mornings and movement behavior reminder notifications in the afternoons. Students agreed with the proposed suggestion of receiving content articles and push notifications on Monday and Wednesday mornings and the movement behavior reminder notifications on Tuesday and Thursday afternoons. These suggested timings were adopted as part of the intervention delivery.

**Figure 2:**
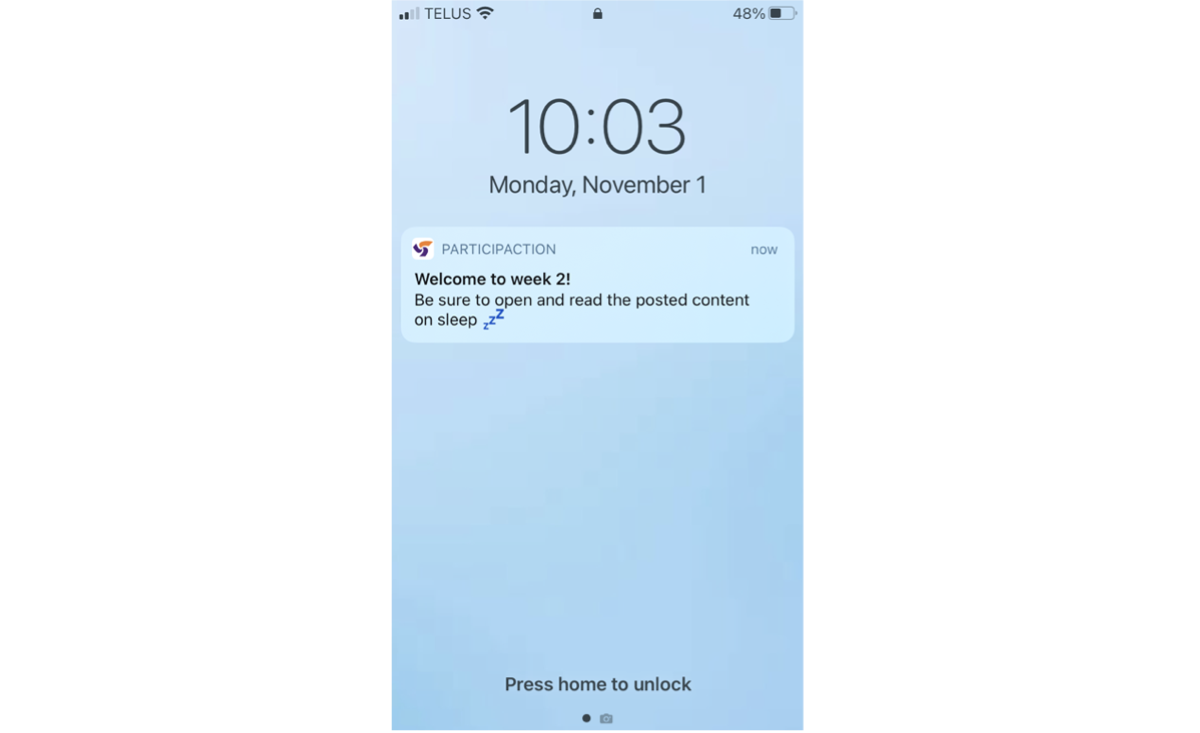
A push notification reminding students to view the new article in the ParticipACTION app.

**Figure 3:**
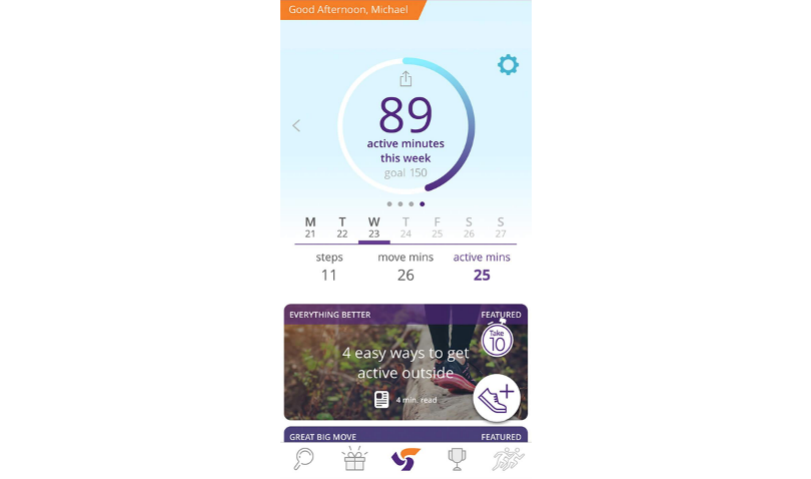
The ParticipACTION app home page showing user active minutes, steps, and move minutes.

**Figure 4:**
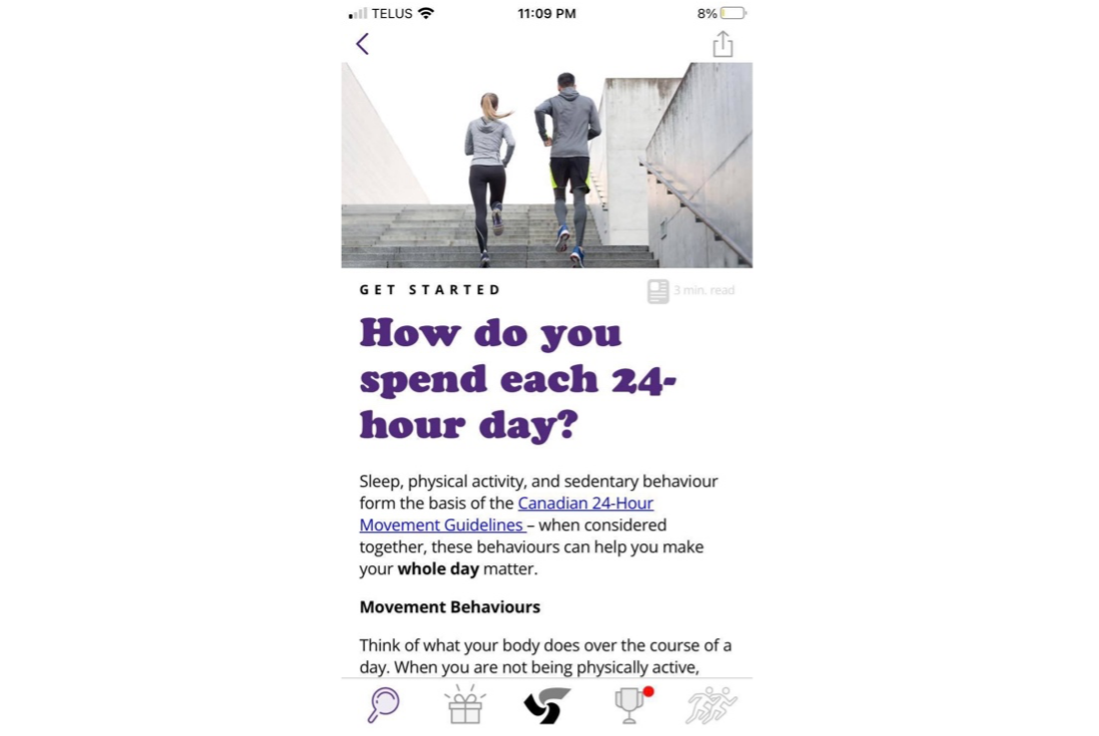
An intervention content article in the ParticipACTION app discussing the 24-Hour Movement Guidelines.

**Figure 5:**
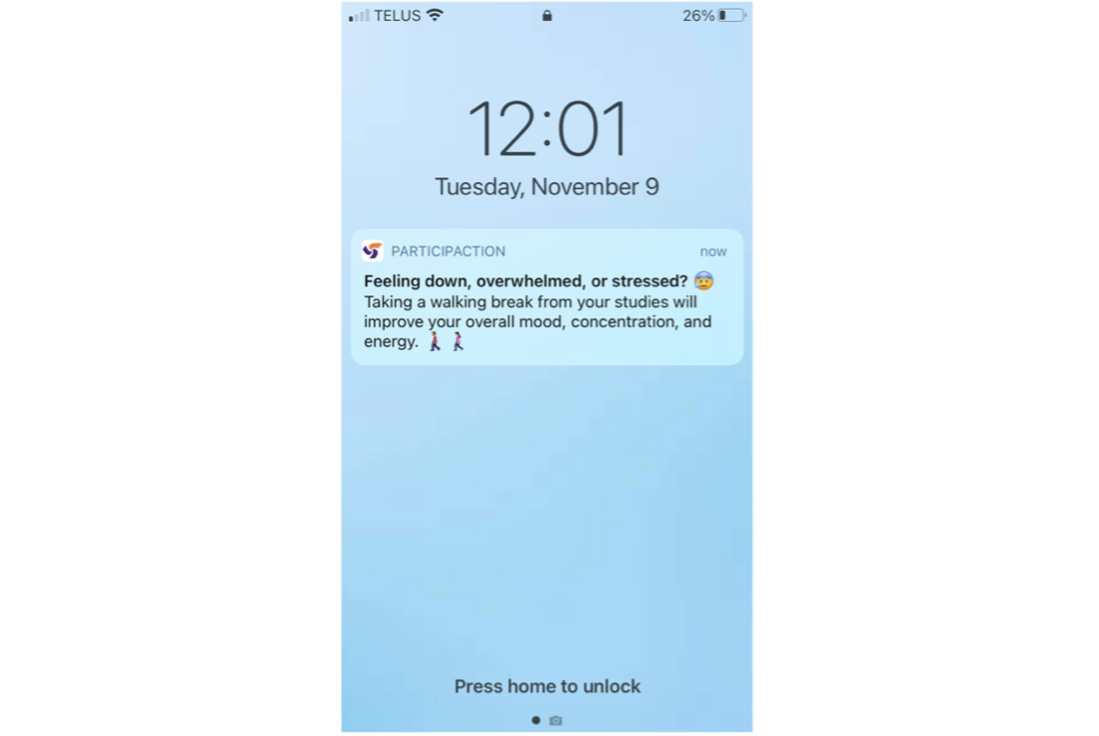
Movement behavior reminder notification delivered to students—reminders focused on a particular movement behavior or mental well-being.

The updated ParticipACTION app content was then reviewed and approved by the entire working group and campus representatives (including first-year students) by email. The content was sent to ParticipACTION staff with expertise in health promotion, knowledge translation, and marketing. The content was reviewed to ensure that ParticipACTION standards, tone, and voice were maintained. Staff edited and formatted the content and returned the content to the knowledge translation lead (JRT), 24HMG project coordinator (SMF), and research assistant (BT), who made the final edits and returned the content to ParticipACTION. Finally, ParticipACTION staff uploaded the content into the ParticipACTION app and prescheduled the delivery of the content articles, push notifications, and movement behavior reminder notifications. Once in the app, the content articles were reviewed by 4 of the working group members (SMF, BT, LMV, and JRT) who suggested final edits relating to formatting and image placement. The final ParticipACTION app content text and images for intervention week 1 can be viewed in Multimedia Appendix 4. Figures 2-5 present examples of students’ views in the ParticipACTION app, including examples of notifications, the app home page, and a content article.

### Step 5: Intervention Implementation Plan (July 2021-September 2021)

To implement and deliver the intervention at the 2 institutions, ParticipACTION staff, recruiters, and study coordinators were required to act as implementers. For this intervention, the adopters were the first-year postsecondary students for whom the intervention was developed.

#### Implementers

ParticipACTION staff were responsible for formatting and uploading the content articles into the ParticipACTION app and for scheduling the release or delivery of the content and notifications. Recruiters (eg, campus staff and lecturers at UBC and QU) at each institution acted as gatekeepers for the intervention. Their support and recruitment efforts were critical for recruiting students to the intervention. The study coordinators (SMF and JM) were responsible for determining student eligibility for participation in the developed intervention and for participant intake processes.

#### Adopters

The intervention adopters included first-year undergraduate postsecondary students from QU and UBC. To participate in the intervention, students had to meet the eligibility criteria determined a priori by the working group and refined during consultations with campus representatives (see the section Step 3: Intervention Design [April 2021-July 2021]). Interested students completed an eligibility screening questionnaire via Qualtrics.

The inclusion criteria for eligibility stipulated that students must (1) be first year, full-time, postsecondary students enrolled in an undergraduate program who have just graduated high school or students who graduated high school and took a gap year before enrolling in their current undergraduate degree and have not attended another college or university in the interim; (2) be born in 2003 or earlier (ie, students who will be turning 18 years or older in 2021, as the behavioral recommendations in the 24HMG for adults are applicable to ages 18-64 years); (3) want to make a change in their physical activity, sedentary behavior, or sleep (ie, are contemplators, intenders, or actors); and (4) not be accumulating 150 minutes per week of aerobic moderate to vigorous intensity physical activity (ie, getting <150 minutes per week of moderate to vigorous intensity physical activity); (5) score between 20 and 60 out of 70 on the Warwick-Edinburgh Mental Wellbeing Scale [34], indicating average or low mental well-being; (6) have a smartphone; (7) feel comfortable navigating technology (ie, students were asked if they felt comfortable navigating the ParticipACTION app via their smartphone and were provided with the ParticipACTION app accessibility specifications); and (8) be able to read, speak, and understand English.

The exclusion criteria stipulated that students would be ineligible if they (1) have a physical impairment, as the intervention content focuses on the 24HMG, which was not designed for persons with a physical disability or impairment and therefore may not be appropriate for these individuals; (2) have an acute concussion or symptoms of postconcussion syndrome, as the intervention content was delivered via smartphone and these individuals should avoid screens; or (3) are a QU student enrolled in the kinesiology undergraduate degree program or are a UBC student enrolled in any of the following courses: HEAL 100, KIN 300, NURS 180, or NURS 290, as these degrees or courses are confounders for the evaluation (ie, students learn intervention content in these courses).

#### Screening and Intake Processes

The 24HMG project coordinator (SMF) reviewed the completed eligibility screening questionnaires to determine student eligibility to participate in the intervention. Students were contacted by QU and UBC study coordinators (SMF and JM, respectively) informing them of whether they were eligible to participate in the intervention. Eligible participants were invited to participate in intake meetings conducted by the coordinators (SMF and JM). To ensure anonymity, all participants were given a new Gmail (Google Inc) account, which was used to create ParticipACTION and Fitbit app accounts during their intake meeting. Once logged into the ParticipACTION app, participants were given the ParticipACTION+ code by the study coordinator, enabling intervention content and notification delivery. Both coordinators were provided with the ParticipACTION+ code as well, allowing them to receive intervention content and notifications to confirm intervention fidelity. During intake, participants were provided with their Inspire 2 Fitbit, asked to log into the Fitbit app using their new Gmail account, and showed how to synchronize their Fitbit to both the Fitbit and ParticipACTION apps. Finally, coordinators also provided a clear, verbal outline of the intervention ParticipACTION content journey and walked through what was included in the PDF resource package and emailed participants the PDF document.

### Step 6: Evaluation Plan (July 2021-Ongoing)

To complete the iterative Intervention Mapping protocol, a plan for the evaluation of the intervention was developed. A nonrandomized proof-of-concept study was conducted to examine the feasibility, acceptability, and potential impact of the app-based intervention. Note that no a priori criteria were set to assess feasibility and acceptability. Data analysis is ongoing. Full details of the evaluation plan will be published in a forthcoming paper; however, a brief summary of the evaluation plan is described here.

Feasibility will be examined by reviewing the recruitment of students to the intervention (eg, the number of students screened and number of students eligible for participation and enrolled in the intervention), and participant attrition over the course of the intervention. Characteristics of participants (ie, demographics including gender, age, ethnicity, etc) were also collected and will be used to describe who participated in the intervention. The feasibility of evaluation measures will be assessed through the evaluation response rates.

Acceptability of the intervention will be assessed by using the data on ParticipACTION app engagement (eg, percentage of participants clicking and engaging with intervention content articles). Satisfaction with the intervention will be evaluated through analysis of a postintervention questionnaire with items measuring participant agreement on whether the intervention was interesting, useful, easy to participate in, credible, and personally important to them.

The evaluation plan also attempted to establish the potential of the intervention for changes in outcomes between baseline and after the intervention. Outcomes of interest included participants’ (1) capability, opportunity, and motivation for the movement behaviors; (2) movement behavior levels; (3) mental well-being; and (4) awareness and knowledge of the 24HMG. Surveys were administered at baseline and after the intervention to assess outcomes 1, 3, and 4. Means and SDs of questionnaire items at each time point and effect sizes between time points will be reported. To assess outcome 2, means and SDs of time spent in each of the movement behaviors before, during, and after the intervention as well as trends in movement behaviors over time will be examined and reported.

### Ethics Approval, Informed Consent, and Participation

Ethics approval for the focus groups conducted with first-year postsecondary students was granted by the QU General Research Ethics Board (GSKHS-329-19). Written informed consent was obtained by all focus group participants. Separate ethics approval was sought and granted by the QU General Research Ethics Board (GSKHS-394-21) and UBC Behavioral Research Ethics Board (H21-02741) for the app-based intervention development, recruitment, data collection, analysis, and reporting activities, including all ParticipACTION and Fitbit app–related components. All participants provided written informed consent for the collection, analysis, and storing of their self-reported (ie, survey) data and ParticipACTION and Fitbit app data. All data were deidentified with identifying information stored in separate, password-protected databases on secure, locked servers and not attached to participants’ study data. Participants who completed the baseline and postquestionnaire survey were compensated with a CAD $5 (US $3.7) e-gift card for each questionnaire. Participants who participated in the intervention were able to keep the provided Fitbit following the intervention period.

## Results

Participant recruitment occurred during September 2021, and the intervention was conducted from October to December 2021. The deadline for completion of the postintervention questionnaire survey by participants was mid-December 2021. We began analyzing the survey, ParticipACTION, and Fitbit data in January 2022, and the publication of the proof-of-concept evaluation is expected in 2023.

## Discussion

### Summary

Given postsecondary students’ suboptimal engagement in the 24HMG behaviors [9], this population is at risk of negative impacts on their physical and mental well-being. In this paper, we describe the application of the Intervention Mapping framework to design and implement an app-based intervention intended to improve first-year postsecondary students’ movement behavior and mental well-being.

### Strengths and Limitations

The design and implementation of the intervention followed an iterative process using several frameworks: the Knowledge-to-Action Framework [6], Intervention Mapping [18,19], the TDF [34,35], and the BCW [21,22]. The steps of Intervention Mapping aligned well with the Knowledge-to Action framework, which has been used to guide the overall 24HMG knowledge translation process [7]. Although Intervention Mapping was used to guide a stepwise intervention design and implementation process, the TDF and BCW enabled the working group to categorize barriers and facilitators and to choose intervention functions and BCTs most likely to overcome barriers and leverage facilitators. When used together, these frameworks were well suited to designing an intervention addressing 3 separate movement behaviors, each of which are complex (ie, not a “single incidence” behavior) and occur in multiple contexts and at various times and frequencies. The involvement of a multidisciplinary team helped ensure that the intervention was designed appropriately for the target population [5,52]. Indeed, meticulous efforts were taken to design the intervention content with multiple layers of consultations.

This intervention has several limitations. First, content articles delivered to students in the ParticipACTION app are in a written format; some students may have benefited from the opportunity to view content in alternative formats such as video, which may also increase engagement with the content. In addition, the selected intervention components lacked the elements of social support. Social support can help to facilitate students’ performance of the movement behaviors and to improve mental well-being—students identified social support as being an important facilitator for movement behavior change within the needs assessment (Multimedia Appendix 2). However, given the context of the COVID-19 pandemic and the uncertainty of the return of on-campus programing, we chose to not engage interventionist support at the onset of intervention design. This consideration also informed the pragmatic decision to implement individual-level strategies to address behavioral change, even though data from the needs assessment in step 1 suggested that institutional-level strategies (eg, encouraging residences to enforce quiet hours) may also promote performance of the movement behaviors in line with the 24HMG recommendations. Institutional-level strategies need to be explored in future research. Despite these limitations, we developed an intervention that could be scalable to other postsecondary campuses in the future. Finally, prompts and push notifications delivered through the ParticipACTION app may have implications for students’ recreational screen time, either through minutes spent on the ParticipACTION app or other unintended app use (eg, students viewing other apps on their phone after being prompted by the ParticipACTION app). However, it is important to consider whether said screen time is considered “productive” or “purposeful” (eg, time spent on the ParticipACTION app may be considered educational as opposed to minutes spent absentmindedly scrolling through a social media app). Although beyond the scope of this study, future work should examine unintended effects of app-based health interventions and recreational screen time and, more broadly, sedentary behavior.

### Conclusions

Intervention Mapping, with the use of the TDF and BCW, was a useful approach to combine evidence and theoretical concepts to guide the design and implementation of a ParticipACTION app–based intervention targeting postsecondary students’ movement behaviors and mental well-being. This study may be used as an example for other researchers developing multiple behavior change app–based interventions, and should the forthcoming proof-of-concept intervention evaluation demonstrate the intervention’s acceptability, feasibility, and potential impact, the intervention may provide a scalable method for improving students’ movement behaviors and mental well-being and future behavior change interventions.

## Appendix

Multimedia Appendix 1 Details of the student intervention working group constituency.

Multimedia Appendix 2 Details of the complete list of performance and change objectives with the combined links between the Theoretical Domains Framework domains (ie, determinants of the movement behaviors) and the Behaviour Change Wheel components.

Multimedia Appendix 3 Details of intervention components and descriptions.

Multimedia Appendix 4 Description of intervention week 1–ParticipACTION app content text and images.

## Abbreviations

24HMG: 24-Hour Movement Guidelines
BCT: behavior change technique
BCW: Behavior Change Wheel
COM-B: capability, opportunity, motivation, behavior
QU: Queen’s University
TDF: Theoretical Domains Framework
UBC: University of British Columbia

## Data Availability

Multimedia Appendix 2 includes the complete list of performance and change objectives, with mapping of the combined links between Theoretical Domains Framework domains (ie, determinants of movement behaviors) and Behavior Change Wheel components. After analysis of the data examining the feasibility, acceptability, and potential impact of the intervention (eg, the survey, ParticipACTION, and Fitbit data) is complete, the data sets will be available from the corresponding author upon reasonable request.
