# Supplementary material for: Development of a ParticipACTION App–Based Intervention for Improving Postsecondary Students’ 24-Hour Movement Guideline Behaviors: Protocol for the Application of Intervention Mapping
Source: JMIR Res Protoc. 2023 Mar 14;12:e39977. doi: 10.2196/39977 (PMC10131646; doi:10.2196/39977)
Supplement: Multimedia Appendix 1 [file resprot_v12i1e39977_app1.docx]

**Multimedia Appendix 1**

**Table S1.** Student intervention working group constituency

| **Member** | **Affiliation** | **Role** |
| --- | --- | --- |
| Stephanie M. Flood, MSc | Project Coordinator, 24-Hour Movement Guidelines for Adults aged 18–64 years and Adults aged 65 years or older, Queen’s University | 24HMG Project Coordinator and Queen’s University Study Coordinator; Co-led Intervention Mapping Steps 1-6; Responsible for intervention eligibility screening of all students and Queen’s University participant intake and communication |
| Brooke Thompson, BSc(c) | Bachelor of Science Candidate, School of Kinesiology and Health Studies, Queen’s University | Research Assistant; Provided insight for Intervention Mapping Steps 3-6; Drafted content articles; Led student consultations and follow-up interviews |
| Guy Faulkner, PhD | Professor, School of Kinesiology, Faculty of Education, University of British Columbia | 24HMG Knowledge Translation Expert; Provided oversight for Intervention Mapping Steps 1-6; Facilitated University of British Columbia campus consultation process; Lead for the University of British Columbia intervention site |
| Leigh M. Vanderloo, PhD | Knowledge Translation Manager, ParticipACTION | ParticipACTION Representative; Provided insight for Intervention Mapping Steps 1-6; Facilitated consultation processes for ParticipACTION app intervention development and delivery |
| Beth Blackett, MA | Health Promotion Special Projects, Student Wellness Services, Queen’s University | Queen’s University Health Promotion Representative; Provided insight for Intervention Mapping Steps 1-6; Facilitated Queen’s University campus consultation process and recruitment strategy process |
| Matt Dolf, PhD | Director, Office of Wellbeing Strategy, University of British Columbia; Chair, Canadian Health Promoting Campuses Network | University of British Columbia Student Wellbeing Representative; Provided insight for Intervention Mapping Steps 1-6; Facilitated University of British Columbia campus consultation process |
| Amy E. Latimer-Cheung, PhD | Professor, School of Kinesiology and Health Studies, Queen’s University | 24HMG Knowledge Translation Expert; Provided insight for Intervention Mapping Steps 1-6 |
| Mary Duggan, CAE | Former Manager, Canadian Society for Exercise Physiology | Canadian Society for Exercise Physiology Representative; Provided insight for Intervention Mapping Steps 1-5 |
| Katie M. Di Sebastiano, PhD | Assistant Professor, Department of Sport and Exercise Sciences, Durham University | ParticipACTION App Evaluation Expert; Provided insight for Intervention Mapping Steps 4-5 pertaining to ParticipACTION app data collection and evaluation |
| Kirstin N. Lane, PhD | Board Representative, Canadian Society for Exercise Physiology; Assistant Teaching Professor, Exercise Science, Physical and Health Education, University of Victoria | Canadian Society for Exercise Physiology Representative; Provided insight for Intervention Mapping Steps 1-6 |
| Melissa C. Brouwers, PhD | Director, School of Epidemiology and Public Health at the Faculty of Medicine, University of Ottawa | 24HMG Knowledge Translation Methodologist; Provided insight for Intervention Mapping Steps 1-6 |
| Julia McKenna, MSc(c) | MSc Candidate, School of Kinesiology, Faculty of Education, University of British Columbia | University of British Columbia Study Coordinator; Provided insight for Intervention Mapping Steps 4-6; Responsible for University of British Columbia participant intake and communication |
| Tala Chulak-Bozzer, MSc | Former Behaviour Insights Manager, ParticipACTION | ParticipACTION Representative; Provided insight for Intervention Mapping Steps 4-6 pertaining to ParticipACTION app data collection and evaluation |
| Daniel Fuller, PhD | Professor, Department of Community Health and Epidemiology, College of Medicine, University of Saskatchewan | Fitbit Data Collection and Analysis Expert; Provided insight for Intervention Mapping Step 6 pertaining to Fitbit evaluation |
| Geralyn R. Ruissen, PhD | PhD, School of Kinesiology, Sport, and Recreation, University of Alberta | ParticipACTION App Evaluation Expert; Provided insight for Intervention Mapping Step 6 pertaining to ParticipACTION app data evaluation |
| Shelby L. Sturrock, PhD(c) | PhD Candidate, Dalla Lana School of Public Health, University of Toronto | Fitbit Data Collection and Analysis Expert; Provided insight for Intervention Mapping Step 6 pertaining to Fitbit evaluation |
| Jennifer R. Tomasone, PhD | Associate Professor, School of Kinesiology and Health Studies, Queen’s University | 24HMG Knowledge Translation Lead; Co-led Intervention Mapping Steps 1-6; Lead for the Queen’s University intervention site |

Note. 24HMG, 24-Hour Movement Guidelines.
