# Supplementary material for: Development of a ParticipACTION App–Based Intervention for Improving Postsecondary Students’ 24-Hour Movement Guideline Behaviors: Protocol for the Application of Intervention Mapping
Source: JMIR Res Protoc. 2023 Mar 14;12:e39977. doi: 10.2196/39977 (PMC10131646; doi:10.2196/39977)
Supplement: Multimedia Appendix 2 [file resprot_v12i1e39977_app2.docx]

**Multimedia Appendix 2**

**Supplementary Table S2.** Complete list of performance and change objectives with the combined links between the Theoretical Domains Framework domains (i.e., determinants of the movement behaviours) and the Behaviour Change Wheel components

| **Performance Objectives** | **Change Objectives** | **Belief Statements**  ***(from Giouridis et al [33])*** | **TDF** | **BCW Components**  **(*from Michie et al [21,22]*)** | | | |
| --- | --- | --- | --- | --- | --- | --- | --- |
|  |  |  |  | **COM-B** | **Intervention Functions** | **Policy Categories** | **Behaviour Change Techniques** |
|  | | | | | | | |
| **P01**. Student performs a variety of types and intensities of physical activity, which includes:   - Moderate to vigorous aerobic physical activities such that there is an accumulation of at least 150 minutes per week - Muscle strengthening activities using major muscle groups at least twice a week - Several hours of light physical activities, including standing | Have the physical capabilities/ strength to engage in certain kinds of recommended physical activity | *Undervalue/ disregard of movement behaviour* (B) I lack sufficient skills to comfortably engage in physical activities. | Physical Skills | Physical Capability | - Training - Enablement | - Communication/ marketing - Guidelines - Fiscal measures - Regulation - Legislation - Service provision - Environmental/ social planning | 4.1 Instruction on how to perform behaviour  6.1 Demonstration of the behaviour  8.1 Behavioural practice/rehearsal  8.7 Graded tasks |
|  | Provide opportunity to observe and engage in physical activity with peers | *Societal norms and trends* (F) I will engage in physical activity as a result of my peers and social media, e.g., seeing posts about striving for the summer body or doing physical activity TikTok challenges with peers. | Social Influences | Social Opportunity | - Environmental restructuring - Enablement - Modelling - Restriction | - Communication/ marketing - Guidelines - Fiscal measures - Regulation - Legislation - Service provision - Environmental/ social planning | 3.1 Social support (unspecified)  6.1 Demonstration of the behaviour  6.2 Social comparison |
|  | **Consider strategies to overcome intimidation at the gym.**  E.g., plan workout ahead of time, exercise during off-peak hours, workout with a friend or mentor | *Social comparison w/ direct environment*  (B) Observing very fit individuals at the gym can be intimidating and make it harder for me to feel comfortable engaging in physical activity. | Social Influences | Social Opportunity | - Environmental restructuring - Enablement - Modelling - Restriction | - Communication/ marketing - Guidelines - Fiscal measures - Regulation - Legislation - Service provision - Environmental/ social planning | 1.2 Problem solving  1.4 Action planning  3.2 Social support (practical)  6.1 Demonstration of the behaviour  8.1 Behaviour practice/rehearsal  8.2 Behaviour substitution  8.3 Habit formation  8.4 Habit reversal |
|  | **Provide low-cost or free options for physical activity** | *Finance* (B) Physical activity costs too much - I can't afford a gym membership and finding free physical activity options can be time consuming.  NOTE: Barrier = especially relevant to UBC students where gym membership not included in tuition | Environmental Context & Resources | Physical Opportunity | - Training - Environmental restructuring - Enablement - Restriction | - Communication/ marketing - Guidelines - Fiscal measures - Regulation - Legislation - Service provision - Environmental/ social planning | 4.1 Instruction on how to perform behaviour  12.1 Restructuring the physical environment  12.5 Adding objects to the environment |
|  | **Encourage taking advantage of good weather to be active and consider getting outside** | *Environmental Factors (weather)* (F) Good weather can positively influence my decision to be physically active | Environmental Context & Resources | Physical Opportunity | - Training - Environmental restructuring - Enablement - Restriction | - Communication/ marketing - Guidelines - Fiscal measures - Regulation - Legislation - Service provision - Environmental/ social planning | 7.1 Prompts/cues  11.3 Conserving mental resources |
|  | Provide options and strategies on how to keep active in bad weather | *Environmental Factors (weather)* (B) Bad weather can negatively influence my decision to be physically active | Environmental Context & Resources | Physical Opportunity | - Training - Environmental restructuring - Enablement - Restriction | - Communication/ marketing - Guidelines - Fiscal measures - Regulation - Legislation - Service provision - Environmental/ social planning | 1.2 Problem solving  4.1 Instruction on how to perform behaviour |
|  | **Believing that engaging in physical activity is beneficial, despite setbacks and discomfort following periods of inactivity** | *Physical state of health* (B) My body hurts after engaging in physical activity following a period of inactivity. | Beliefs about Consequences | Reflective Motivation | - Education - Persuasion - Incentivisation - Coercion | - Communication/ marketing - Guidelines - Fiscal measures - Regulation - Legislation - Service provision | 4.1 Instruction on how to perform behaviour  5.1 Information about health consequences  5.2 Salience of consequences  5.6 Information about emotional consequences  8.1 Behavioural practice/rehearsal  9.2 Pros and cons  9.3 Comparative imagining of future outcomes  10.1 Material incentive (behaviour)  10.7 Self-incentive  10.8 Incentive (outcome)  10.9 Self-reward  10.10 Reward (outcome) |
|  | Rethink and redevelop goals related to engagement in physical activity, refocus motivation (minimizing emphasis on physique). | *Societal norms and trends* (F) Meeting the physical activity recommendations is important because I want to look like the fitness influencers I see on social media.  *Social comparison within direct environment* (F) I will prioritize meeting the physical activity recommendations over the sleep recommendations as I can visibly see outcomes of engaging in physical activity (i.e., an improved physique). | Goals | Reflective Motivation | - Education - Persuasion - Incentivisation - Coercion | - Communication/ marketing - Guidelines - Fiscal measures - Regulation - Legislation - Service provision | 1.1 Goal setting (behaviour)  1.2 Problem solving  1.3 Goal setting (outcome)  1.4 Action planning  1.5 Review behaviour goal(s)  1.6 Discrepancy between current behaviour and goal  1.7 Review outcome goal(s)  1.8 Behavioural contract  2.3 Self-monitoring of behaviour  8.7 Graded tasks  10.2 Material reward (behaviour)  10.4 Social reward  10.8 Self-reward |
|  | **Discuss how engaging in physical activity can improve mood, while emphasizing importance of finding activities that individuals enjoy** | *Undervalue/disregard of movement behaviour* (B) I don't enjoy or like physical activity (e.g., exercising or going to the gym). | Emotion | Automatic Motivation | - Persuasion - Incentivisation - Training - Environmental restructuring - Enablement - Modelling - Coercion | - Communication/ marketing - Guidelines - Fiscal measures - Regulation - Legislation - Service provision - Environmental/ social planning | 2.4 Self-monitoring of outcomes of behaviour  5.6 Information about emotional consequences  9.3 Comparative imagining of future outcomes  11.2 Reduce negative emotions  13.2 Framing/reframing  15.3 Focus on past successes |
|  | | | | | | | |
| **P02**. Student gets 7 to 9 hours of good-quality sleep on a regular basis, with consistent bed and wake-up times | Identify positive role models (e.g., family) that engage in healthy sleep habits and enlist them for support | *Family - Social influences/obligations*  (F) My family (their behaviours and/or beliefs) influences my meeting the sleep recommendation (e.g., going to bed at a reasonable time at night) | Social Influences | Social Opportunity | - Environmental restructuring - Enablement - Modelling - Restriction | - Communication/ marketing - Guidelines - Fiscal measures - Regulation - Legislation - Service provision - Environmental/ social planning | 3.1 Social support (unspecified)  3.2 Social support (practical)  6.1 Demonstration of the behaviour  6.2 Social comparison  6.3 Information about others' approval |
|  | **Provide instruction and strategies on how to improve sleep quality (e.g., sleep hygiene) and aim for consistent bed and wake up times** | *Transition from high school* (B) The inconsistency of my schedule from day to day impacts my ability to have a consistent bed and wake-up times.  *Casual Employment*  (B) Having to work late or overnight for my job impacts my sleep. | Environmental Context & Resources | Physical Opportunity | - Training - Environmental restructuring - Enablement - Restriction | - Communication/ marketing - Guidelines - Fiscal measures - Regulation - Legislation - Service provision - Environmental/ social planning | 1.2 Problem solving  3.2 Social support (practical)  4.1 Instruction on how to perform behaviour  7.1 Prompts/cues  7.5 Remove aversive stimulus  12.1 Restructuring the physical environment  12.2 Restructuring the social environment  12.3 Avoidance/reducing exposure to cues for the behaviour  12.5 Adding objects to the environment |
|  | Encourage residences to enforce quiet hours and promote an atmosphere that encourages healthy sleep habits | *Residential Environment*  (F) A quiet living environment (including quiet or respectful roommates/floormates) helps me to meet the sleep recommendations  (B) A loud living environment (including loud or noise-producing roommates/floormates) can prevent me from meeting the sleep recommendations | Environmental Context & Resources | Physical Opportunity | - Training - Environmental restructuring - Enablement - Restriction | - Communication/ marketing - Guidelines - Fiscal measures - Regulation - Legislation - Service provision - Environmental/ social planning | 3.2 Social support (practical)  12.1 Restructuring the physical environment  12.2 Restructuring the social environment  12.5 Adding objects to the environment |
|  | | | | | | | |
| **P03**. Student limits sedentary time to 8 hours or less, which includes:   - No more than 3 hours of recreational screen time - Breaking up long periods of sitting as often as possible | Create individual and group identity that limiting/monitoring recreational screen time is important and part of a healthy lifestyle for students and the current generation | *Societal norms and trends* (B) It's normal for people in my generation, including students, to have high amounts of recreational screen time, so I don't need to try to meet the sedentary behaviour recommendations. | Professional/ Social Role & Identity | Reflective Motivation | - Education - Persuasion - Incentivisation - Coercion | - Communication/ marketing - Guidelines - Fiscal measures - Regulation - Legislation - Service provision | 3.1 Social support (unspecified)  6.2 Social comparison  9.1 Credible source  13.2 Framing/ reframing  13.5 Identity associated with changed behaviour |
|  | **Believing that not engaging in screen time can be relaxing and still feel like downtime** | Downtime/ relaxation (B) Limiting my recreational screen time to meet the recommendations would negatively impact my relaxation time | Beliefs about Consequences | Reflective Motivation | - Education - Persuasion - Incentivisation - Coercion | - Communication/ marketing - Guidelines - Fiscal measures - Regulation - Legislation - Service provision | 4.1 Instruction on how to perform behaviour  5.1 Information about health consequences  5.2 Salience of consequences  5.3 Information about social and environmental consequences  5.6 Information about emotional consequences  9.2 Pros and cons  9.3 Comparative imagining of future outcomes  10.1 Material incentive (behaviour)  10.7 Self-incentive  10.8 Incentive (outcome)  10.9 Self-reward  10.10 Reward (outcome) |
|  | | | | | | | |
| **P04**. Student replaces sedentary behaviour with additional physical activity and trades light physical activity for more moderate to vigorous physical activity, while preserving sufficient sleep, to achieve greater health benefits. | **Have an awareness and understanding of the existing Movement Behaviour Guidelines** | *Initial Perceptions* (F) There are Guidelines which lay out clear recommendations (to meet) for physical activity, sedentary behaviour, and sleep | Knowledge | Psychological Capability | - Education - Training - Enablement | - Communication/ marketing - Guidelines - Fiscal measures - Regulation - Legislation - Service provision - Environmental/ social planning | 4.1 Instruction on how to perform behaviour |
|  | **Know how the movement behaviours are connected to one another other.**  i.e., how changes in one behaviour impact the other Guideline behaviours | *Understanding interconnectedness of behaviours* (F) It is important to know the evidence/rationale behind the recommendations and how the behaviours are interconnected | Knowledge | Psychological Capability | - Education - Training - Enablement | - Communication/ marketing - Guidelines - Fiscal measures - Regulation - Legislation - Service provision - Environmental/ social planning | 4.1 Instruction on how to perform behaviour |
|  | **Have an awareness of the benefits of performing the movement behaviours** | *Societal norms and trends* (F) Awareness of the benefits of performing the movement behaviours is helpful. | Knowledge | Psychological Capability | - Education - Training - Enablement | - Communication/ marketing - Guidelines - Fiscal measures - Regulation - Legislation - Service provision - Environmental/ social planning | 5.1 information about health consequences  5.3 Information about social and environmental consequences |
|  | **Have an awareness of the facilities and equipment (i.e., resources) that exist on campus and are available to students that support the movement behaviours** | *Resources* (F) Awareness of available campus resources helps with meeting the Guideline recommendations | Knowledge | Psychological Capability | - Education - Training - Enablement | - Communication/ marketing - Guidelines - Fiscal measures - Regulation - Legislation - Service provision - Environmental/ social planning | 4.1 Instruction on how to perform behaviour |
|  | **Knowing how to perform the movement behaviours and knowing strategies on how to meet the movement behaviour recommendations** | *Skills and knowledge* (F) Awareness of how to perform the movement behaviours and specific strategies on how to meet the movement behaviour recommendations are helpful. | Knowledge | Psychological Capability | - Education - Training - Enablement | - Communication/ marketing - Guidelines - Fiscal measures - Regulation - Legislation - Service provision - Environmental/ social planning | 2.2 Feedback on behaviour  4.1 Instruction on how to perform behaviour  8.1 Behavioural practice/rehearsal  8.2 Behaviour substitution |
|  | **Have the ability to develop and maintain a schedule to perform the movement behaviours** | *Skills and Knowledge* (F) Can meet the recommendations by actively learning and executing skills and strategies to perform the movement behaviours (e.g., scheduling, alarms/reminders, cues)  *Established routine* (F) The ability to create and implement a schedule is an important skill for meeting the Guideline recommendations  *Transition from high school* (B) Not having the ability to maintain some form of a consistent daily routine/schedule negatively impacts performance of behaviours  (F) The ability to learn how to create and implement a consistent daily routine/schedule in university is an important skill for meeting the Guideline recommendations | Skills | Psychological Capability | - Education - Training - Enablement | - Communication/ marketing - Guidelines - Fiscal measures - Regulation - Legislation - Service provision - Environmental/ social planning | 1.4 Action planning  4.1 Instruction on how to perform behaviour |
|  | **Remembering to perform the movement behaviours, working to incorporate behaviours into routine - making them second nature/habit** | *Value of movement behaviours* (F) Remembering to perform the movement behaviours is easy once they have become second nature or habit.  *Time management* (F) Incorporating the movement behaviours into my daily routine, or making them habit, allows me to meet the Guideline recommendations without having to think about it much. | Memory, Attention, and Decision Processes | Psychological Capability | - Education - Training - Enablement | - Communication/ marketing - Guidelines - Fiscal measures - Regulation - Legislation - Service provision - Environmental/ social planning | 1.9 Commitment  7.1 Prompts/cues  8.1 Behavioural practice/ rehearsal  8.2 Behaviour substitution  8.3 Habit formation  8.4 Habit reversal  11.3 Conserving mental resources |
|  | **Improve ability to make the movement behaviours a priority** | *Undervalue (disregard) of movement behaviours* (B) There are many distracting priorities as a student and the movement behaviours can 'fall to the back burner' (or get pushed to the side).  *Internal motivation* (B) There's no deadline to perform the movement behaviours, i.e., it's not mandatory to perform the movement behaviours unlike academics. | Memory, Attention, and Decision Processes | Psychological Capability | - Education - Training - Enablement | - Communication/ marketing - Guidelines - Fiscal measures - Regulation - Legislation - Service provision - Environmental/ social planning | 1.9 Commitment  7.1 Prompts/cues  8.1 Behavioural practice/ rehearsal  8.2 Behaviour substitution  11.3 Conserving mental resources |
|  | **Make decisions over tasks/ activities that can be conducted while performing the movement behaviours (e.g., studying, socializing) or that preserve performance of the movement behaviours.** | *Transition from High School* (B) I will choose to engage in other activities that I enjoy or prioritize more (i.e., social or academic activities) rather than choose to engage in the movement behaviours.  *Academic obligations* (B) I will often decide to not perform the movement behaviour(s) because my academic obligations take precedence over meeting the Guideline recommendations. | Memory, Attention, and Decision Processes | Psychological Capability | - Education - Training - Enablement | - Communication/ marketing - Guidelines - Fiscal measures - Regulation - Legislation - Service provision - Environmental/ social planning | 1.9 Commitment  7.1 Prompts/cues  8.1 Behavioural practice/ rehearsal  8.2 Behaviour substitution  11.3 Conserving mental resources  12.1 Restructuring the physical environment  12.2 Restructuring the social environment |
|  | **Know how to reduce screen time in the evening and make the decision to prioritize sleep - how to avoid 'revenge bedtime procrastination'** | *Societal norms and trends* (B) I will consciously make the decision to put off sleep and rather spend time on my phone - resulting in a decreased amount of sleep time. | Memory, Attention, and Decision Processes | Psychological Capability | - Education - Training - Enablement | - Communication/ marketing - Guidelines - Fiscal measures - Regulation - Legislation - Service provision - Environmental/ social planning | 1.9 Commitment  7.1 Prompts/cues  7.5 Remove aversive stimulus  8.1 Behavioural practice/ rehearsal  8.2 Behaviour substitution  12.1 Restructuring the physical environment |
|  | **Develop skills of action-planning, self-monitoring of behaviour(s), and breaking existing habits (e.g., being sedentary for long periods of time, or staying up late)**  E.g., monitoring and planning may include reminders, alarms, notes, filling water bottle, scheduling and/or establishing a routine - perhaps with a friend. | *Skills and Knowledge* (F) I use prompts to help perform the movement behaviours; I'll set reminders/alarms on my phones, write sticky notes and/or drink water when studying so that I have to get up to refill the cup/bottle.  *Established routine* (F) Establishing a routine, which may include scheduling and maintaining consistency, helps with my adherence to performing the movement behaviours.  *Internal motivation* (F) Scheduling when I will perform the behaviours helps me to feel motivated and work towards meeting the Guideline recommendations.  *Social Influences - Friends* (F) Performing physical activity with a friend (e.g., a workout buddy) helps me to meet the physical activity recommendations. | Behavioural Regulation | Psychological Capability | - Education - Training - Enablement | - Communication/ marketing - Guidelines - Fiscal measures - Regulation - Legislation - Service provision - Environmental/ social planning | 1.1 Goal setting (behaviour)  1.2 Problem solving  1.4 Action Planning  2.3 Self-monitoring of behaviour  4.1 Instruction on how to perform the behaviour  7.1 Prompts/cues  8.1 Behavioural practice/rehearsal  8.2 Behaviour substitution  8.3 Habit formation  8.4 Habit reversal  11.3 Conserving mental resources  12.5 Adding objects to the environment |
|  | **Consider having a buddy, partner or creating a group for peer support and encouragement for performing the movement behaviours** | *Friends - Social influences/obligations*  (F) I am encouraged to meet the Guideline recommendations when I have the social support of a peer to perform the movement behaviours. | Social Influences | Social Opportunity | - Environmental restructuring - Enablement - Modelling - Restriction | - Communication/ marketing - Guidelines - Fiscal measures - Regulation - Legislation - Service provision - Environmental/ social planning | 3.1 Social support (unspecified)  6.2 Social comparison  12.2 Restructuring the social environment  13.1 Identification of self as role model |
|  | **Consider engaging in friendly competition with a peer(s) - to work toward, meet or exceed one or more of the movement behaviours** | Social comparison w/ direct environment  (F) I compare my behaviours to my peers, and I strive to be the 'best', resulting in me working toward, meeting, or exceeding the Guideline recommendations. | Social Influences | Social Opportunity | - Environmental restructuring - Enablement - Modelling - Restriction | - Communication/ marketing - Guidelines - Fiscal measures - Regulation - Legislation - Service provision - Environmental/ social planning | 1.1 Goal setting (behaviour)  1.4 Action planning  3.2 Social support (practical)  6.2 Social comparison  12.2 Restructuring the social environment  13.1 Identification of self as role model |
|  | **Provide opportunity to observe peers/individuals meeting the movement behaviours (i.e., positive role models);** Encourage residences to promote and support student engagement in movement behaviours | Residential *environment*  (F) My roommate's/floormate's positive movement behaviour habits can influence me to also perform the movement behaviours and create positive habits.  (B) My roommate’s/ floormate's negative movement behaviour habits can influence me, negatively impacting whether I perform the movement behaviours | Social Influences | Social Opportunity | - Environmental restructuring - Enablement - Modelling - Restriction | - Communication/ marketing - Guidelines - Fiscal measures - Regulation - Legislation - Service provision - Environmental/ social planning | 3.1 Social support (unspecified)  3.2 Social support (practical)  6.1 Demonstration of the behaviour  6.2 Social comparison |
|  | Encourage professors to support in-lecture breaks and to actively cue students to stand/walk/stretch;  Encourage students to alter or rethink how they utilize their workspaces | *Environmental Factors*  (F) I will break up my sedentary time when my professor provides a break in lecture, and they encourage us to stand up and stretch rather than sit. | Social Influences | Social Opportunity | - Environmental restructuring - Enablement - Modelling - Restriction | - Communication/ marketing - Guidelines - Fiscal measures - Regulation - Legislation - Service provision - Environmental/ social planning | 3.2 Social support (practical)  6.1 Demonstration of the behaviour  6.3 Information about others' approval |
|  | **Identify how connecting with family and performing the movement behaviours can be incorporated together (e.g., standing while facetiming) or how to still engage family while preserving performance of the movement behaviours (e.g., set a time limit for phone or videocalls to ensure bedtimes are maintained, try to find times to connect earlier in the day)** | *Family - Social influences/obligations*  (B) In order to stay in touch with my family, I must connect with them virtually and typically at night, negatively influencing my ability to meet the recreational screen time and sleep recommendations. | Social Influences | Social Opportunity | - Environmental restructuring - Enablement - Modelling - Restriction | - Communication/ marketing - Guidelines - Fiscal measures - Regulation - Legislation - Service provision - Environmental/ social planning | 1.1 Goal setting (behaviour)  1.2 Problem solving  1.4 Action planning  3.1 Social support (unspecified)  8.1 Behaviour practice/ rehearsal  8.2 Behaviour substitution  8.3 Habit formation  8.4 Habit reversal  12.2 Restructuring the social environment  13.1 Identification of self as role model |
|  | **Identify how socializing or connecting with peers/friends and performing the movement behaviours can be incorporated together or how to still engage in important social activities while preserving performance of the movement behaviours.**  E.g., Only go out x2 nights a week, limit # of drinks, set a time you have to leave/log off, stand while facetiming rather than sitting | *Transition from high school* (B) Consistently being surrounded by my peers can negatively influence my ability to meet the Guideline recommendations; I feel peer pressure to engage in social activities and not perform healthy behaviours (e.g., sleep).  *University culture - Social influences/ obligations* (B) I can be influenced to stay out late and party with my peers, negatively impacting my sleep and ability to engage in physical activity the following day.  *Downtime/relaxation*  (B) My friends can negatively influence the amount of screen time I engage in (e.g., time spent facetiming or watching videos together)  *Societal norms and trends* (B) I feel peer pressure to engage in 'normal' social activities, such as connecting with peers late at night to socialize with friends/partners who may live at different institutions or in different time zones, preventing my sleep and increasing my screen time.  *Friends - Social influences/obligations*  (B) My friends can negatively influence by ability to meet the recreational screen time and sleep recommendations; they encourage me to stay up late and spend time virtually socializing/ connecting. | Social Influences | Social Opportunity | - Environmental restructuring - Enablement - Modelling - Restriction | - Communication/ marketing - Guidelines - Fiscal measures - Regulation - Legislation - Service provision - Environmental/ social planning | 1.1 Goal setting (behaviour)  1.2 Problem solving  1.4 Action planning  3.1 Social support (unspecified)  8.1 Behaviour practice/rehearsal  8.2 Behaviour substitution  8.3 Habit formation  8.4 Habit reversal  12.2 Restructuring the social environment  13.1 Identification of self as role model |
|  | **Promote use and access of existing campus resources and opportunities for engagement in the movement behaviours (including free resources/ opportunities)** | *Value of movement behaviour* (F) Resources and opportunities on campus help me to engage in the various movement behaviours, for example, being able to access the gym, intramurals, standing desks and even stairs help to facilitate some form of physical activity.  *Resources*  QU (B) - The physical activity resources that exist on campus aren't promoted well by the university; we need an introduction or description of what is offered/available at the ARC (e.g., a virtual tour).  UBC (B) - There are resources on campus to help break up sedentary time (e.g., desk cycles, standing desks), but they aren't promoted well by the university.  *Finance* (F) Having access to and being aware of free physical activity options helps me be active. | Environmental Context & Resources | Physical Opportunity | - Training - Environmental restructuring - Enablement - Restriction | - Communication/ marketing - Guidelines - Fiscal measures - Regulation - Legislation - Service provision - Environmental/ social planning | 4.1 Instruction on how to perform behaviour  7.1 Prompts/cues |
|  | **Promote and provide instruction on the use of equipment/tools (e.g., alarm, reminders on trackers/watches, calendars) to cue performance of movement behaviours** | Skills and Knowledge (F) Using equipment, such as an alarm, calendar, or watch, is helpful for meeting the Guideline recommendations | Environmental Context & Resources | Physical Opportunity | - Training - Environmental restructuring - Enablement - Restriction | - Communication/ marketing - Guidelines - Fiscal measures - Regulation - Legislation - Service provision - Environmental/ social planning | 4.1 Instruction on how to perform behaviour  7.1 Prompts/cues  11.3 Conserving mental resources  12.5 Adding objects to the environment |
|  | **Provide instruction on how to perform behaviours in ways that can be incorporated into current routines and schedules** | *Internal Motivation* (B) I lack the time, due to the amount of work I must complete in a week, to engage in the movement behaviours.  *Transition from high school* (F) Having a break in between my classes gives me time to be physically active, breaking up my sedentary time.  *Traveling* (B) Travel can impact my ability to perform the movement behaviours adequately. | Environmental Context & Resources | Physical Opportunity | - Training - Environmental restructuring - Enablement - Restriction | - Communication/ marketing - Guidelines - Fiscal measures - Regulation - Legislation - Service provision - Environmental/ social planning | 1.2 Problem solving  3.2 Social support (practical)  4.1 Instruction on how to perform behaviour  7.1 Prompts/cues  11.3 Conserving mental resources  12.2 Restructuring the social environment  12.3 Avoidance/reducing exposure to cues for the behaviour |
|  | **Identify strategies (e.g., standing up and stretching during in-lecture breaks; standing while studying) and promote campus resources (e.g., where standing desks and deskcycles can be accessed) that help reduce sedentary behaviour and increase light physical activity.**  Encourage professors to support in-lecture breaks and to actively cue students to stand/walk/stretch; encourage universities to provide more standing desks and desk cycles for use | *Academic Obligations* (B) The set-up of campus does not make engaging in the movement behaviours easy - lecture halls and study spaces typically facilitate being sedentary.  *Environmental Factors* (B) The campus environment can discourage engaging in the movement behaviours - lecture halls and study spaces typically facilitate being sedentary. | Environmental Context & Resources | Physical Opportunity | - Training - Environmental restructuring - Enablement - Restriction | - Communication/ marketing - Guidelines - Fiscal measures - Regulation - Legislation - Service provision - Environmental/ social planning | 4.1 Instruction on how to perform behaviour  7.1 Prompts/cues  11.3 Conserving mental resources  12.1 Restructuring the physical environment  12.5 Adding objects to the environment |
|  | **Identify and encourage use of strategies to break existing phone/technology habits** | *Societal norms and trends* (B) Having a phone (or other technology) negatively impacts my recreational screen time, sleep quantity and quality. | Environmental Context & Resources | Physical Opportunity | - Training - Environmental restructuring - Enablement - Restriction | - Communication/ marketing - Guidelines - Fiscal measures - Regulation - Legislation - Service provision - Environmental/ social planning | 1.2 Problem solving  3.2 Social support (practical)  4.1 Instruction on how to perform behaviour  7.1 Prompts/cues  7.5 Remove aversive stimulus  11.3 Conserving mental resources  12.1 Restructuring the physical environment  12.2 Restructuring the social environment  12.3 Avoidance/reducing exposure to cues for the behaviour |
|  | **Provide strategies on how to break up sedentary time while commuting (e.g., standing up on public transport, walking part of the journey)** | *Commuting* (B) Having to commute to campus impacts my movement behaviours; I get less physical activity, spend more time being sedentary and I get less sleep (I have to wake up early to commute to class) | Environmental Context & Resources | Physical Opportunity | - Training - Environmental restructuring - Enablement - Restriction | - Communication/ marketing - Guidelines - Fiscal measures - Regulation - Legislation - Service provision - Environmental/ social planning | 1.2 Problem solving  4.1 Instruction on how to perform behaviour  7.1 Prompts/cues  11.3 Conserving mental resources |
|  | **Highlight ways that physical activity is already incorporated into existing activities (e.g., active transport, standing on your feet at work or in lab) and how to increase levels further** | *Environmental Factors* (F) The campus environment can help to encourage healthy movement behaviours, where I have to walk to get to class or I have to stand in a lab.  *Commuting* (F) Living in walking distance to campus impacts physical activity - I walk everywhere and I'm more likely to go to the gym since it's nearby.  *Casual Employment*  (F) Having a job that involves being active (e.g., being on your feet, walking, lifting) helps me to increase my light physical activity and simultaneously decrease my sedentary behaviour. | Environmental Context & Resources | Physical Opportunity | - Training - Environmental restructuring - Enablement - Restriction | - Communication/ marketing - Guidelines - Fiscal measures - Regulation - Legislation - Service provision - Environmental/ social planning | 4.1 Instruction on how to perform behaviour  7.1 Prompts/cues  11.3 Conserving mental resources |
|  | Create individual and group identity that engaging in the movement behaviours is part of a healthy student lifestyle and is linked with improvements in academics and can be achieved while maintaining a social life | *Academic Obligations* (B) As a student, I should prioritize my academic obligations over these movement behaviours - there isn't time to meet the movement behaviour recommendations.  *Transition from high school* (B) Taking on the role of student, and the overwhelming nature of new university experiences - such as academic and social responsibilities - make it infeasible to engage in the movement behaviours as recommended  *University culture - social influence/ obligation* QU (B) Trying to meet the movement behaviour recommendations would get in the way of socializing or partying; it's normal for Queen's students to prioritize participation in the party culture. | Professional/ Social Role & Identity | Reflective Motivation | - Education - Persuasion - Incentivisation - Coercion | - Communication/ marketing - Guidelines - Fiscal measures - Regulation - Legislation - Service provision | 3.1 Social support (unspecified)  6.2 Social comparison  9.1 Credible source  13.2 Framing/ reframing |
|  | **Believing in ability to meet the movement Guidelines may require building the confidence toward meeting one recommendation, as working towards all at once may be overwhelming** | *Understanding interconnectedness of behaviours* (F) Confident can meet the Guideline recommendations (perform the target behaviours), by first adequately meeting one behaviour recommendation.  *Initial perceptions* (B) Confidence is hindered, based on the belief that it is difficult to meet the recommendations for all three behaviours at the same time. | Beliefs About Capabilities | Reflective Motivation | - Education - Persuasion - Incentivisation - Coercion | - Communication/ marketing - Guidelines - Fiscal measures - Regulation - Legislation - Service provision | 1.1 Goal setting (behaviour)  1.2 Problem solving  1.4 Action planning  2.7 Feedback on outcome(s) of behaviour  4.1 Instruction on how to perform behaviour  8.1 Behaviour practice/rehearsal  8.7 Graded tasks  10.4 Social reward  15.1 Verbal persuasion about capability  15.2 Mental rehearsal of successful performance  15.3 Focus on past success  15.4 Self-talk |
|  | **Have a strong will and belief can perform the movement behaviours** | *Value of movement behaviour* (F) I am confident that I can perform the movement behaviours because the behaviours are important to me  *Undervalue/disregard of movement behaviour* (B) It's difficult to perform the movement behaviours in line with the Guideline recommendations because I lack the competence and/or self-efficacy  *Internal motivation* (F) I am confident in performing the movement behaviours when I'm motivated.  (B) It's difficult to perform the movement behaviours when I'm unmotivated or feel burned out.  *Transition from high schoo*l (F) Confident in ability to engage in healthy movement behaviours as I have freedom of choice of when and how I can perform these behaviours.  *Family - social obligations* (F) It's easier for me to meet the Guidelines because I have been performing the behaviour(s) from a young age or I have observed a parent performing the behaviour(s) as recommended.  *Environmental factors (weather)* (F) My self-efficacy to be physically active increases when the weather is good  (B) My self-efficacy to be physically active decreases when the weather is bad  *Commuting* (F) I am more likely to meet the Guideline recommendations by living on or near campus (e.g., easier to visit the gym and engage in more incidental light physical activity such as walking)  *Family - social obligations* (B) It's difficult for me to meet Guideline recommendations because my family needs are more important to me. | Beliefs About Capabilities | Reflective Motivation | - Education - Persuasion - Incentivisation - Coercion | - Communication/ marketing - Guidelines - Fiscal measures - Regulation - Legislation - Service provision | 1.1 Goal setting (behaviour)  1.2 Problem solving  1.4 Action planning  4.1 Instruction on how to perform behaviour  8.1 Behaviour practice/rehearsal  8.7 Graded tasks  10.4 Social reward  15.1 Verbal persuasion about capability  15.2 Mental rehearsal of successful performance  15.3 Focus on past success  15.4 Self-talk |
|  | **Believing that meeting the movement guidelines are achievable will require self-discipline and self-regulation skills** | *Skills and knowledge* (B) It’s difficult to meet the sedentary behaviour recommendations because you have to actively cue yourself to break up sitting or limit your sedentary time.  *Established routine* (F) I am capable of meeting the Guideline recommendations when I have an organized schedule.  *Transition from high school* (F) Confident in ability to engage in healthy movement behaviours as I have freedom of choice of when and how I can perform these behaviours.  (B) I'm not capable of performing the movement behaviours because I have difficulty managing my time efficiently.  *Travel* (B) It's difficult for me to be able to perform the movement behaviours in line with the guidelines when I travel  *Transition from high school* (B) I'm not capable of performing the movement behaviours because I have difficulty managing my time efficiently.  *Physical state of health* (B) My self-efficacy to engage in physical activity is low if I have been inactive for a while.  *Friends - social obligations* (B) It's difficult to meet the recreational screen time recommendations because I socialize with friends virtually.  *Societal norms and trends* (B) I feel like I need to be on my phone to stay connected to peers, friends, family, the news - I can't limit my recreational screen time to just 3 hours.  (B) It's difficult to meet the sleep recommendations because I am on my phone or computer before bed, and I lose track of the time. | Beliefs About Capabilities | Reflective Motivation | - Education - Persuasion - Incentivisation - Coercion | - Communication/ marketing - Guidelines - Fiscal measures - Regulation - Legislation - Service provision | 1.1 Goal setting (behaviour)  1.2 Problem solving  1.4 Action planning  4.1 Instruction on how to perform behaviour  8.1 Behaviour practice/rehearsal  8.3 Habit formation  8.7 Graded tasks  10.4 Social reward  15.1 Verbal persuasion about capability  15.2 Mental rehearsal of successful performance  15.3 Focus on past success  15.4 Self-talk |
|  | **Believing that meeting the movement guidelines is achievable will require recognition that behaviours can be adopted in such a way that they can fit into one’s busy schedule** | *Time management* (B) I can't meet the Guideline recommendations; it's too time consuming to meet all of the individual recommendations.  *Academic obligations* (B) My self-efficacy for performing the movement behaviours is low because of competing academic priorities.  *Casual employment* (B) When I have to work late for my job I cannot get enough sleep.  *University culture - social influence* QU (B) My self-efficacy for engaging in physical activity and adequate sleep is low; I lack time due to my competing, higher priorities of socializing and partying. | Beliefs About Capabilities | Reflective Motivation | - Education - Persuasion - Incentivisation - Coercion | - Communication/ marketing - Guidelines - Fiscal measures - Regulation - Legislation - Service provision | 1.2 Problem solving  1.4 Action planning  4.1 Instruction on how to perform behaviour  6.1 Demonstration of the behaviours  8.1 Behaviour practice/rehearsal  8.7 Graded tasks  15.1 Verbal persuasion about capability  15.2 Mental rehearsal of successful performance  15.3 Focus on past success  15.4 Self-talk |
|  | Being able to make the whole day matter even when some aspects are outside of the students' control | *Mental health impacts* (B) I can't get adequate sleep because of stress; my sleep quality is out of my control  *Residential environment* (B) My sleep quality is out of my control; residence neighbours stop by to visit or people in the building are loud.  *Commuting* (B) It's more difficult to meet the Guideline recommendations by living off-campus and having to commute; commuting results in decreased time and increased tiredness preventing engagement in behaviours. | Beliefs About Capabilities | Reflective Motivation | - Education - Persuasion - Incentivisation - Coercion | - Communication/ marketing - Guidelines - Fiscal measures - Regulation - Legislation - Service provision | 1.2 Problem solving  8.1 Behaviour practice/rehearsal  8.7 Graded tasks  15.1 Verbal persuasion about capability  15.2 Mental rehearsal of successful performance  15.3 Focus on past success  15.4 Self-talk |
|  | **Believing that performing the movement behaviours is beneficial (including more short-term, immediate benefits)** | *Value of movement behaviour* (F) Meeting the Guideline recommendations for the movement behaviours will lead to positive effects: feeling more energized, improved mental health and wellbeing.  *Undervalue/disregard of movement behaviour* (B) The risks associated with not meeting the movement behaviour recommendations aren't relevant to me right now, they're something to worry about when I'm older.  *Skills and knowledge* (F) Meeting the guideline recommendations results in positive health-related effects, e.g., having more energy.  *Nutrition* (F) Decreasing sedentary time (e.g., by studying at a standing desk) and/or increasing physical activity helps to improve metabolism and allows me to burn extra calories.  *Internal motivation* (F) Performing the movement behaviours helps me to feel more motivated and have the energy needed for daily tasks.  *Transition from high school* (F) Meeting the Guideline recommendations would prevent negative outcomes I want to avoid, for example, getting sufficient sleep prevents me from feeling tired the next day.  *Societal norms and trends* (F) Meeting the physical activity and sleep recommendations would help prevent negative consequences I want to avoid (e.g., not engaging in physical activity or sleep may lead to me looking unfit or feeling exhausted, respectively).  *Physical state of health* (F) Engaging in physical activity (i.e., safe, physiotherapy activities) helps my body heal after experiencing a physical injury. | Beliefs About Consequences | Reflective Motivation | - Education - Persuasion - Incentivisation - Coercion | - Communication/ marketing - Guidelines - Fiscal measures - Regulation - Legislation - Service provision | 5.1 Information about health consequences  5.2 Salience of consequences  5.3 Information about social and environmental consequences  5.6 Information about emotional consequences  9.2 Pros and cons  9.3 Comparative imagining of future outcomes  10.1 Material incentive (behaviour)  10.7 Self-incentive  10.8 Incentive (outcome)  10.9 Self-reward  10.10 Reward (outcome) |
|  | **Believing that meeting one of the movement behaviour recommendations or that working towards meeting any of the recommendations is beneficial (i.e., provides health benefits)** | *Understanding interconnectedness of behaviours* (F) Achieving or meeting one behaviour recommendation will lead to enablement of meeting the other behaviour recommendations.  (B) Not achieving/ meeting one behaviour recommendation may lead to/is associated with not meeting the other behaviour recommendations. | Beliefs About Consequences | Reflective Motivation | - Education - Persuasion - Incentivisation - Coercion | - Communication/ marketing - Guidelines - Fiscal measures - Regulation - Legislation - Service provision | 5.1 Information about health consequences  5.2 Salience of consequences  5.3 Information about social and environmental consequences  5.6 Information about emotional consequences  9.2 Pros and cons  9.3 Comparative imagining of future outcomes  10.1 Material incentive (behaviour)  10.7 Self-incentive  10.8 Incentive (outcome)  10.9 Self-reward  10.10 Reward (outcome) |
|  | Believing that family or people of importance think you should engage in performing the movement behaviours | *Family - social obligations* (F) My family would be disappointed in me if I weren't to engage in healthy movement behaviours | Beliefs About Consequences | Reflective Motivation | - Education - Persuasion - Incentivisation - Coercion | - Communication/ marketing - Guidelines - Fiscal measures - Regulation - Legislation - Service provision | 6.3 Information about others' approval  9.1 Credible source  9.2 Pros and cons |
|  | **Believing that meeting the movement Guidelines is feasible while meeting academic priorities and can benefit academics.** | *Transition from high school* (B) If I were to dedicate my time to meeting the Guideline recommendations, my academics would suffer.  *Time management* (B) If I were to meet all of the Guideline recommendations my other priorities (e.g., studying) may be negatively affected.  *Academic obligations* (B) If I engaged in the movement behaviours as recommended I wouldn't have enough time to focus on work; my studies would suffer. | Beliefs About Consequences | Reflective Motivation | - Education - Persuasion - Incentivisation - Coercion | - Communication/ marketing - Guidelines - Fiscal measures - Regulation - Legislation - Service provision | 4.1 Instruction on how to perform behaviour  5.1 Information about health consequences  5.2 Salience of consequences  5.3 Information about social and environmental consequences  5.6 Information about emotional consequences  8.1 Behavioural practice/ rehearsal  9.2 Pros and cons  9.3 Comparative imagining of future outcomes  10.1 Material incentive (behaviour)  10.7 Self-incentive  10.8 Incentive (outcome)  10.9 Self-reward  10.10 Reward (outcome) |
|  | **Believing that meeting the movement Guidelines is feasible to achieve while still preserving important social activities.** | *Family - social obligations* (B) Engaging in the movement behaviours would impact my social time with family  *Friends - social influences/obligations* (B) If I met the recreational screen time recommendation it would negatively impact my time spent socializing/ connecting with friends, which is done virtually  *Friends - social influences/obligations* (B) Meeting the sleep recommendations would decrease the amount of time I have available to spend talking with friends/family  *University culture - social obligations* QU (B) Engaging in the movement behaviours (e.g., sleep and physical activity) would inhibit my ability to participate in pivotal social events (e.g., homecoming and social activities which are all key parts of Queen's university culture)  *Residential environment* (B) Engaging in the movement behaviours would lead to missing out on socializing and partying within residence. | Beliefs About Consequences | Reflective Motivation | - Education - Persuasion - Incentivisation - Coercion | - Communication/ marketing - Guidelines - Fiscal measures - Regulation - Legislation - Service provision | 4.1 Instruction on how to perform behaviour  5.1 Information about health consequences  5.2 Salience of consequences  5.3 Information about social and environmental consequences  5.6 Information about emotional consequences  8.1 Behavioural practice/rehearsal  9.2 Pros and cons  9.3 Comparative imagining of future outcomes  10.1 Material incentive (behaviour)  10.7 Self-incentive  10.8 Incentive (outcome)  10.9 Self-reward  10.10 Reward (outcome) |
|  | **Develop intentions and make commitments to perform the behaviours (even when lacking motivation)** | *Internal motivation* (F) I am inclined to engage in healthy movement behaviours when I am feeling motivated, which may include feeling mentally prepared, and/or organized. (B) I am not inclined to engage in healthy movement behaviours when I'm not motivated (e.g., I'm procrastinating or I'm exhausted). | Intentions | Reflective Motivation | - Education - Persuasion - Incentivisation - Coercion | - Communication/ marketing - Guidelines - Fiscal measures - Regulation - Legislation - Service provision | 1.1 Goal setting (behaviour)  1.4 Action planning  1.9 Commitment  5.1 Information about health consequences  9.3 Comparative imagining of future outcomes  10.8 Incentive (outcome)  13.1 Identification of self as role model  13.4 Valued self-identify |
|  | **Develop intentions to perform the movement behaviours, even when not feeling physically or mentally well - recognizing the wellness benefits felt from performing the movement behaviours at any level (e.g., improvement in mood, mental health) and try different strategies** | *Mental Health impacts* (B) I am not inclined to perform the movement behaviours when I am experiencing poor mental health.  *Physical State of Health* (B) I am not inclined to be physically active when I do not feel well. | Intentions | Reflective Motivation | - Education - Persuasion - Incentivisation - Coercion | - Communication/ marketing - Guidelines - Fiscal measures - Regulation - Legislation - Service provision | 1.1 Goal setting (behaviour)  1.2 Problem solving  1.4 Action planning  1.9 Commitment  5.1 Information about health consequences  9.3 Comparative imagining of future outcomes  10.8 Incentive (outcome)  13.1 Identification of self as role model  13.4 Valued self-identify |
|  | **Develop goals to perform the movement behaviours - contemplate how these goals can be tailored and fit with other academic and social priorities** | *Value of movement behaviour* (F) Movement behaviours are a high priority.  *Undervalue/disregard of movement behaviour* (B) Performing the movement behaviours is of low importance or priority to me; spending time on my academics is more productive.  *Transition from high school* (B) Performing the movement behaviours are not a priority to me; they conflict with my other priorities (e.g., social activities, academics).  *Time management* (F) I'm motivated to make time for the movement behaviours that are important to me. (B) Meeting the Guideline recommendations are a low priority; other areas of my life take a higher priority (e.g., studying).  *Academic obligations* (B) Performing the movement behaviours conflict with my academic goals; my movement behaviours are a lower priority.  *Family - social influences/obligations* (B) Engaging in sleep as outlined in the recommendations is low priority to me; spending time connecting or talking with my family is a higher priority.  *Friends - social influences/obligations* (B) The sleep and recreational screen time recommendations are a low priority to me; spending time connecting or talking with friends is more important.  *University culture - social influences/ obligations* (B) Performing the movement behaviours are a low priority to me in comparison to socializing/partying.  *Societal norms and trends* (B) Performing the movement behaviours are a low priority to me in comparison to other activities I prioritize (e.g., time spent on my phone staying connected or up to date on social media).  *Physical state of health* (F) Engaging in physical activity is important to me because it helps my body heal after injury; meaning I can return to my normal physical activity routine. | Goals | Reflective Motivation | - Education - Persuasion - Incentivisation - Coercion | - Communication/ marketing - Guidelines - Fiscal measures - Regulation - Legislation - Service provision | 1.1 Goal setting (behaviour)  1.2 Problem solving  1.3 Goal setting (outcome)  1.4 Action planning  1.5 Review behaviour goal(s)  1.6 Discrepancy between current behaviour and goal  1.7 Review outcome goal(s)  1.8 Behavioural contract  2.3 Self-monitoring of behaviour  8.7 Graded tasks  10.2 Material reward (behaviour)  10.4 Social reward  10.8 Self-reward |
|  | Develop goals focusing on personal incentives/ rewards to encourage performing the movement behaviours | *Value of movement behaviour* (F) I strive to engage in the recommended amounts of each movement behaviour as it is rewarding - it makes you feel energized and good.  *Established routine* (F) Engaging in physical activity or getting adequate sleep feels like a reward; I'm more productive and have more energy.  *Social comparison within direct environment* (F) Engaging in physical activity is rewarding as it results in visible changes to my physique (i.e., how I look) that I value. | Reinforcement | Automatic Motivation | - Persuasion - Incentivisation - Training - Environmental restructuring - Enablement - Modelling - Coercion | - Communication/ marketing - Guidelines - Fiscal measures - Regulation - Legislation - Service provision | 1.1 Goal setting (behaviour)  2.2 Feedback on behaviour  3.1 Social support (unspecified)  7.1 Prompts/cues  9.3 Comparative imagining of future outcomes  10.1 Material incentive (behaviour)  10.2 Material reward (behaviour)  10.4 Social reward  10.9 Self-reward  13.1 identification of self as role model  13.2 Framing/reframing |
|  | Develop and reinforce routines and habits for performing the movement behaviours - contemplate how these habits can be incorporated or fit with other activities | *Friends - social influence/obligations* (B) If I were to perform the movement behaviours as recommended (i.e., sleep and recreational screen time) I would lose time I value spending on my academics and socializing.  *Downtime/relaxation* (B) If I were to perform the movement behaviours as recommended (i.e., sleep and recreational screen time) I would lose the downtime/relaxation time I value. | Reinforcement | Automatic Motivation | - Persuasion - Incentivisation - Training - Environmental restructuring - Enablement - Modelling - Coercion | - Communication/ marketing - Guidelines - Fiscal measures - Regulation - Legislation - Service provision | 1.1 Goal setting (behaviour)  2.2 Feedback on behaviour  3.1 Social support (unspecified)  7.1 Prompts/cues  8.1 Behavioural practice/ rehearsal  8.3 Habit formation  9.3 Comparative imagining of future outcomes  10.1 Material incentive (behaviour)  10.2 Material reward (behaviour)  10.4 Social reward  10.9 Self-reward  13.1 Identification of self as role model  13.2 Framing/reframing |
|  | **Discuss how performing movement behaviours - sleep and physical activity - can improve mood.** | *Value of movement behaviour* (F) I enjoy engaging in physical activity and sleep | Emotion | Automatic Motivation | - Persuasion - Incentivisation - Training - Environmental restructuring - Enablement - Modelling - Coercion | - Communication/ marketing - Guidelines - Fiscal measures - Regulation - Legislation - Service provision | 2.4 Self-monitoring of outcomes of behaviour  5.6 Information about emotional consequences  9.3 Comparative imagining of future outcomes  11.2 Reduce negative emotions  13.2 Framing/reframing  15.3 Focus on past success |
|  | | | | | | | |

*Note*. (B) indicates belief statements that are barriers, while (F) indicates belief statements that are facilitators. BCW, Behaviour Change Wheel; COM-B, Capability, Opportunity, Motivation and Behaviour Framework; TDF, Theoretical Domains Framework. Bolded change objectives indicate change objectives selected by the working group for inclusion within the intervention.
