# Supplementary material for: Development of a ParticipACTION App–Based Intervention for Improving Postsecondary Students’ 24-Hour Movement Guideline Behaviors: Protocol for the Application of Intervention Mapping
Source: JMIR Res Protoc. 2023 Mar 14;12:e39977. doi: 10.2196/39977 (PMC10131646; doi:10.2196/39977)
Supplement: Multimedia Appendix 3 [file resprot_v12i1e39977_app3.docx]

**Multimedia Appendix 3**

**Supplementary Table S3.** Intervention Components and Descriptions

| ***Intervention Component*** | ***Intervention Component Description*** | ***COM-B Components*** | ***Intervention Functions*** | ***Selected BCTs*** | ***Rationale*** |
| --- | --- | --- | --- | --- | --- |
| **Included Intervention Components*^a^*** | | | | | |
| ***Resource Package*** | | | | | |
| 24HMG: ParticipACTION App Study Resource Package | -Participants provided with a resource package including resources for the Canadian 24HMG for Adults (e.g., 24HMG recommendations and infographics), as well as campus, community and web-based resources for physical activity, sedentary behaviour, sleep, and mental health and wellbeing. Resources included free and low-cost options.  -Resources intended to educate participants on how to perform the movement behaviours, enable participants to have the opportunity to engage in the movement behaviours (e.g., workout videos), and provide suggestions on how to restructure environments to encourage the movement behaviours (e.g., designate screen-free zones in the house).  -Resources tailored for each campus and respective city. | Psychological capability  Physical opportunity | Education  Enablement  Environmental restructuring | 4.1 Instruction on how to perform behaviour  6.1 Demonstration of the behaviour  12.5 Adding objects to the environment | STUDENT FOCUS GROUP FEEDBACK^c^  -Need an awareness of the facilities and equipment that exist on campuses  EVIDENCE  -Access to resources and recreational opportunities (including awareness of resources and opportunities) can greatly influence health behaviours (Furlano et al., 2021; Zaidlin et al., 2020) |
| ***ParticipACTION App – Intervention Components*** | | | | | |
| 24HMG ParticipACTION App Study Content Articles | -Twelve succinct content articles, tailored for postsecondary students, developed to take participants on a 6-week content journey to improve their movement behaviours and mental wellbeing.  -Articles educate participants on 24HMG  recommendations, and aim to educate, persuade, and enable participants to perform the movement behaviours by including content focusing on behaviour change strategies (i.e., self-monitoring, goal setting, action planning, self-monitoring of outcomes, and coping planning).  -Articles provide suggestions on how to restructure environments to encourage movement behaviours (e.g., makeshift standing desk), and on how to be active with housemates and friends.  -Links within articles to videos that provide step-by-step instructions on how to perform physical activity.  -Articles educate participants on how performing the movement behaviours as recommended in the 24HMG can improve mental wellbeing.  -Content articles focused on the individual movement behaviours (i.e., physical activity, sedentary behaviour, and sleep) as well as the integrated nature of the movement behaviours.  -The first article in each week focused on giving information about the behaviour. The second article in each week focused on providing a behaviour change strategy in the context of the behavioural focus for that week. The following outlines the presentation of the content:  Week 1: Introduction to the 24HMG  Behaviour change strategy: self-monitoring  Week 2: Sleep  Behaviour change strategy: goal setting  Week 3: Sedentary behaviour  Behaviour change strategy: action planning  Week 4: Light physical activity  Behaviour change strategy: self-monitoring of outcomes  Week 5: Moderate to vigorous physical activity  Behaviour change strategy: coping planning  Week 6: Integrated behaviours  Behaviour change strategy: review of all above strategies | Physical capability  Psychological capability  Physical opportunity  Social opportunity  Reflective motivation  Automatic motivation | Education Persuasion  Training Enablement  Environmental restructuring | 1.1 Goal setting (behaviour)  1.2 Problem solving  1.3 Goal setting (outcome)  1.4 Action planning  1.6 Discrepancy between current behaviour and goal  1.7 Review outcome goal(s)  2.3 Self-monitoring of behaviour  4.1 Instruction on how to perform behaviour  5.1 Information about health consequences  6.1 Demonstration of the behaviour  7.1 Prompts/cues  8.1 Behavioural practice/rehearsal  8.2 Behaviour substitution  8.7 Graded tasks  12.1 Restructuring the physical environment  12.2 Restructuring the social environment  12.5 Adding objects to the environment | STUDENT FOCUS GROUP FEEDBACK^c^  -Need greater knowledge and understanding of the 24HMG, of the benefits of movement behaviours, and how to perform the behaviours – including strategies and tips (e.g., planning, use of reminders)  -Require belief in ability to meet the 24HMG, confidence to perform the movement behaviours, and belief that performing the movement behaviours is beneficial  -Require ability to plan to perform the movement behaviours  -Need to know how to restructure the environment to encourage the movement behaviours (both physically and socially)  EVIDENCE  -SMART goal setting, problem solving, and self-monitoring are useful BCTs for all movement behaviour change (AAFP, 2018)  -E- and m-health interventions aimed at increasing physical activity and decreasing sedentary behaviour should include action planning, coping planning, and self-monitoring, along with combinations of other BCTs (Schroé et al., 2020)  -Multiple behaviour change interventions have shown to be associated with more effective outcomes versus targeting single behaviour interventions (Sweet & Fortier, 2010; Johnson, Paiva, & Cummins, 2008)  -When presenting multiple behaviours to an individual, there is debate regarding which behaviour change should be prioritized or if an individual should attempt to change all behaviours at the same time (James et al., 2016); accordingly, certain content articles focus on the link among the health behaviours while others focus more selectively on a single movement behaviour. |
| Push Notifications | -Content articles were made available to participants twice a week (i.e., Mondays and Wednesdays), accordingly push notifications delivered to participants informing them about the new content in the app every Monday and Wednesday morning. | Automatic motivation | Persuasion  Environmental restructuring | 7.1 Prompts/cues  12.5 Adding objects to the environment | STUDENT FOCUS GROUP FEEDBACK^c^  -N/A  EVIDENCE  -Push notifications improve mobile app engagement (Freyne et al., 2017), session count, time consumption of app content, retention within the app (Pham et al., 2016) |
| Movement Behaviour Reminder Notifications | -Customized notifications delivered to participants encouraging them to engage in a specific movement behaviour. For example, to encourage participants to take a break from sedentary time, they were sent a notification stating, “As you study or work on that assignment, take time to stand and stretch!”.  -Two movement behaviour reminder notifications sent to participants each week (i.e., Tuesday and Thursday afternoons) for a total of 12 notifications delivered over the 6-week intervention. | Automatic motivation | Persuasion  Environmental restructuring | 7.1 Prompts/cues  12.5 Adding objects to the environment | STUDENT FOCUS GROUP FEEDBACK^c^  -Need reminders to perform the movement behaviours, with aim to incorporate behaviours into routine.  -Need reminders that engaging in sleep and physical activity can improve mood.  EVIDENCE  -App reminders/prompts can be important for the user to remember to perform the intended behaviour(s), while ensuring to not over prompt users (Dennison et al., 2013) |
| **Excluded Intervention Components^b^** | | | | | |
| ***ParticipACTION App*** | | | | | |
| Content Article Engagement Badges | -Participants would receive a badge within the app for clicking and engaging with the two content articles each week of the intervention. Participants would be able to earn a total of six badges.  -Badge motivates participants to click and engage with intervention content, and rewards participants with additional entries in draws for prizes within the app. | Automatic motivation | Persuasion  Incentivization | 2.3 Self-monitoring of behaviour  10.2 Material reward (behaviour) | STUDENT FOCUS GROUP FEEDBACK^c^  -N/A  EVIDENCE  -Badges act as a completion-contingent reward (Deci et al., 1999) which may motivate task completion (Neupane et al., 2021) |
| Within Campus Competition | -Participants attending the same institution would be put into teams and compete with one another, to see who has the highest step count or the most move minutes.  -Participants would be able to view scores on a leaderboard to see how fellow participants at their institution were performing.  -The team with the highest step count or most move minutes at the end of the intervention would win a $50 e-gift card for each participant. | Social opportunity  Automatic motivation | Incentivization  Enablement  Modelling | 2.3 Self-monitoring of behaviour  6.2 Social comparison  10.2 Material reward (behaviour) | STUDENT FOCUS GROUP FEEDBACK^c^  -Friendly competition with peers can help to work toward, meet or exceed the physical activity recommendation.  -The opportunity to view others physical activity behaviour can be motivating (e.g., positive role models).  EVIDENCE  -Physical activity apps that enable  competition with friends by ranking or earning rewards can motivate students to engage in physical activity (Middelweerd et al., 2015)  -Receiving an award can be perceived as motivational by students and as input for a competition with friends (Middelweerd et al., 2015) |
| Between Campus Competition | -Participants at each institution would compete to see which institution can collectively accumulate more steps by the end of the 6-week intervention.  -Weekly updates would be provided to notify participants of each institution’s progress.  -Participants at the institution with the most steps at the end of the 6-week intervention would each receive a $10 e-gift card. | Social opportunity  Automatic motivation | Incentivization  Enablement  Modelling | 2.3 Self-monitoring of behaviour  6.2 Social comparison  10.2 Material reward (behaviour) | STUDENT FOCUS GROUP FEEDBACK^c^  -Friendly competition with peers can help to work toward, meet or exceed the physical activity recommendation.  -The opportunity to view others physical activity behaviour can be motivating (e.g., positive role models).  EVIDENCE  -Physical activity apps that enable  competition with friends by ranking or earning rewards can motivate students to engage in physical activity (Middelweerd et al., 2015)  -Receiving an award can be perceived as motivational by students and as input for a competition with friends (Middelweerd et al., 2015) |

Note. 24HMG, 24-Hour Movement Guidelines; COM-B, Capability, Opportunity, Motivation of Behaviour; N/A, not applicable; TDF, Theoretical Domains Framework; BCT, behaviour change technique.

^a^Within Step 3: Intervention Design see the “Intervention components: Practical Applications and Mode of Delivery” and “Refining Intervention Components” sections for additional details regarding intervention component selection and refinement

^b^Intervention components were excluded following discussion by partners and the working group as the proposed intervention components would not function as intended due to limitations of ParticipACTION app functionality; within Step 3: Intervention Design see the “Refining Intervention Components” section for more details

^c^Full focus group findings are published elsewhere (Giouridis et al. [33])
