# Supplementary material for: Development of a ParticipACTION App–Based Intervention for Improving Postsecondary Students’ 24-Hour Movement Guideline Behaviors: Protocol for the Application of Intervention Mapping
Source: JMIR Res Protoc. 2023 Mar 14;12:e39977. doi: 10.2196/39977 (PMC10131646; doi:10.2196/39977)
Supplement: Multimedia Appendix 4 [file resprot_v12i1e39977_app4.docx]

**Multimedia Appendix 4**

**Intervention Week 1 - ParticipACTION App Content Text and Images**

**Week 1 – Introduction to the 24-Hour Movement Guidelines**

**Notification 1: Reminder** (sent Monday, October 25^th^ @ 10am)

- Headline: Your whole day matters! ⏰
- Body: Be sure to check the app for this week’s introduction to the Canadian 24-Hour Movement Guidelines.

**Article 1:** *Available in app Monday, October 25^th^ at 12am*


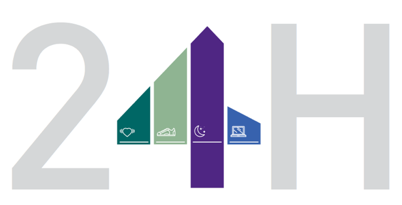


How do you spend each 24-hour day?

Sleep, physical activity, and sedentary behaviour form the basis of the [Canadian 24-Hour Movement Guidelines](https://www.participaction.com/en-ca/benefits-and-guidelines/adults-18-to-64) – when considered together, these behaviours can help you make your **whole day** matter.

**Movement Behaviours**

Think of what your body does over the course of a day. When you are not being physically active, you’ll likely say you’re not moving - instead you may be sitting, lying down or you may be sleeping. Over the course of a 24-hour period, we all engage in a mix of movement behaviours and these behaviours exist on a continuum from low to high energy expenditure. Sleep is at the low-end of the energy continuum while physical activity is at the high-end.

- **Sleep** is a regenerative behaviour and you expend very little energy while you sleep.
- When you are awake and lying down, reclining, or sitting and not moving very much, you are engaging in **sedentary behaviour**. If you are sitting in a chair or laying on your bed while reading this, you are being sedentary and expending slightly more energy than if you were sleeping.
- If you were to stand or move your body while lying down, reclining, or sitting your energy expenditure would increase and you would now be performing **physical activity.** And as you move your body more, you increase the intensity of physical activity from light, to moderate, to vigorous.


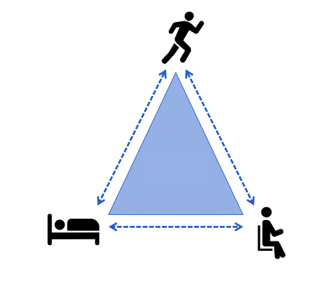


**Connection between the 24-Hour Movement Behaviours**

We have all experienced a time where we have not had a good night sleep and as a result, we feel lethargic and tired for the rest of the day. Getting physical activity on days we have not had enough sleep can be challenging, and we may default to more sedentary behaviours. However, spending time on a screen while being sedentary affects our ability to fall asleep, which means another night of insufficient sleep, and so the cycle can continue.

Sleep, physical activity and sedentary behaviour are all connected, and time spent in one behaviour impacts the time spent in the other two behaviours. Making a change, even a small change, in one behaviour will impact your other two behaviours.

For example, if you were to swap 30 minutes of watching an episode of your favourite show on a streaming channel (e.g., Netflix, Crave, Prime, etc.) with a leisurely walk around campus or your neighbourhood (i.e., swapping sedentary time for some light physical activity) you would be moving more, reducing sedentary time, and setting yourself up for a better sleep.


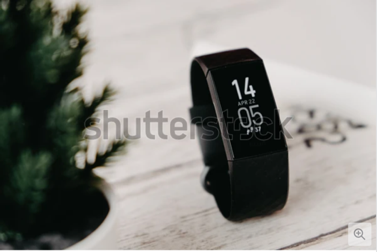


One way to determine how much time you spend performing each behaviour is to use a fitness tracker (i.e., a Fitbit) [*INSERT Hyperlink: Link to ParticipACTION app video “How to sync your fitness tracker”*] or time-use diary to monitor your behaviour. Monitoring gives you an opportunity to think about what options you may have to move more, reduce sedentary time, and thus, sleep well. You can also log how you feel during or after performing a behaviour which can serve as a source of reflection and motivation.

What small changes can you do to make your whole day matter?

**Notification 2: Message** *(sent Tuesday, October 26^th^ @ 1pm)*

- Headline: Get that physical activity in!
- Body: Go outside for a walk today even for 10 minutes – every move counts! 👟🚶🏻‍♀️

**Notification 3: Reminder** *(sent Wednesday, October 27^th^ @ 10am)*

- Headline: You’re almost through week one! 🎊 ✅
- Body: Open the app to learn more about making your whole day matter.

**Article 2:** *Available in app Wednesday, October 27^th^ at 12am*


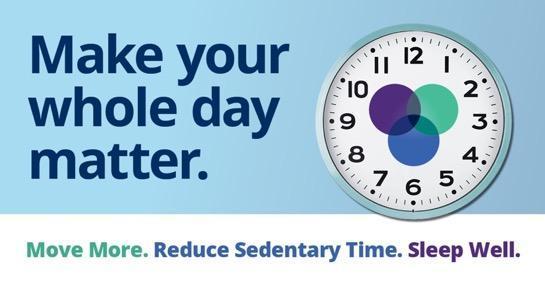


What do the 24-Hour Movement Guidelines mean for you?

The Canadian 24-Hour Movement Guidelines offer recommendations for how you can make your whole day matter. According to the guidelines, taking advantage of every day means moving more, reducing sedentary time, and sleeping well.


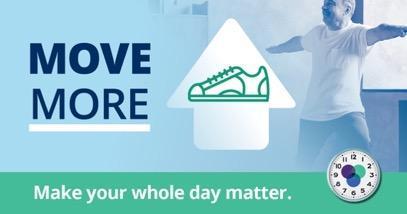


The Guidelines specify that **moving more** involves adding movement throughout your day, including a variety of types and intensities of physical activity:

- At least 150 minutes of moderate to vigorous aerobic physical activity per week.
- Muscle strengthening activities at least twice a week.
- Several hours of light physical activity, including standing.

Moving more can mean many things and doesn’t need to be complicated! You will learn more about the different types of physical activity in the coming weeks but for now, keep the following examples in mind:

You might choose to perform **light physical activity** by doing things such as:

- Walking leisurely
- Stretching
- Standing

You can engage in **moderate-to-vigorous aerobic physical activity** by:

- Playing a sport (i.e., volleyball or soccer)
- Jogging
- Waking briskly
- Biking

Or you can engage in **muscle strengthening** activities by:

- Using your body weight to do a movement (push-ups)
- Working against resistance like dumbbells or resistance bands (e.g., squats, lunges)

Try these YMCA workout videos to get a feel for each intensity level! [*INSERT Hyperlink: Link to PAC app video “Try these YMCA workout videos”]*


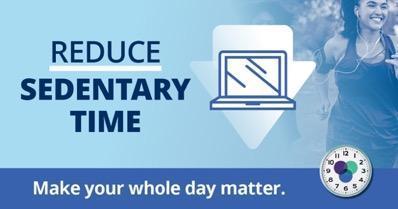


**Reducing sedentary behaviour** involves limiting sedentary time to 8 hours or less and also includes:

- No more than 3 hours of recreational screen time per day
- Recreational screen time includes the time outside of schoolwork that you watch programs and videos, text, use social media, browse the web, and play video games.
- Breaking up long periods of sitting as often as possible

Reducing sedentary behaviour can be achieved by engaging in physical activity, creating a standing or active workspace, or setting time limits or alarms to limit your recreational screen time.


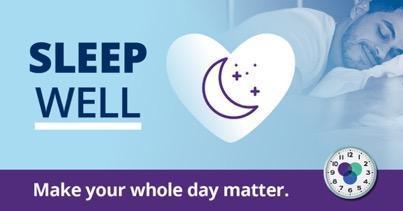


**Sleeping well** involves setting yourself up for 7 to 9 hours of good-quality sleep on a regular basis, with consistent bed and wake-up times. Keeping a consistent schedule means getting up and going to bed at a similar time every day, even on weekends! Sleeping well can help you feel energized to engage in physical activity and reduce sedentary time.


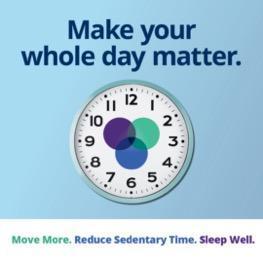


Moving more, reducing sedentary time, and sleeping well can result in short-term benefits such as elevated mood, a positive outlook, increased ability to focus, which can translate to improvements in academic or work performance. Long term benefits include decreased risk of weight gain and improvements in anxiety, depression, cognition and quality of life. Exceeding the minimum physical activity guideline recommendations (i.e., increasing physical activity by more than 150 minutes a week) will provide even greater health benefits.

Remember that beginning to change a behaviour can be difficult for many people but progressing towards any of the Guideline recommendations, even by making small changes, will result in some health benefits.

What is one thing you can try today to move more, sleep well, or reduce sedentary time?

Some activity is always better than none – every move counts!

**Notification 4: Message** *(sent Thursday, October 28^th^ @ 1pm)*

- Headline: Need a mental refresh?
- Body: As you study or work on that assignment, take time to stand and stretch! 🙆🏽‍♀️🙆🏼‍♂️
